# A Novel Resistance Pathway for Calcineurin Inhibitors in the Human-Pathogenic Mucorales Mucor circinelloides

**DOI:** 10.1128/mBio.02949-19

**Published:** 2020-01-28

**Authors:** Sandeep Vellanki, R. Blake Billmyre, Alejandra Lorenzen, Micaela Campbell, Broderick Turner, Eun Young Huh, Joseph Heitman, Soo Chan Lee

**Affiliations:** a South Texas Center for Emerging Infectious Diseases (STCEID), Department of Biology, The University of Texas at San Antonio, San Antonio, Texas, USA; b Department of Molecular Genetics and Microbiology, Duke University Medical Center, Durham, North Carolina, USA; University of Texas Health Science Center

**Keywords:** *Mucor*, mucormycosis, amino acid permease, calcineurin, dimorphism, drug resistance mechanisms, protein kinase A

## Abstract

Mucormycosis is an emerging lethal fungal infection in immunocompromised patients. Mucor circinelloides is a causal agent of mucormycosis and serves as a model system to understand genetics in Mucorales. Calcineurin is a conserved virulence factor in many pathogenic fungi, and calcineurin inhibition or deletion of the calcineurin regulatory subunit (CnbR) in *Mucor* results in a shift from hyphal to yeast growth. We analyzed 36 calcineurin inhibitor-resistant or bypass mutants that exhibited hyphal growth in the presence of calcineurin inhibitors or in the yeast-locked *cnbR*Δ mutant background without carrying any mutations in known calcineurin components. We found that a majority of the mutants had altered sequence in a gene, named here *bycA* (bypass of calcineurin). *bycA* encodes an amino acid permease. We verified that both the *bycA*Δ single mutant and the *bycA*Δ *cnbR*Δ double mutant are resistant to calcineurin inhibitor FK506, thereby demonstrating a novel mechanism of resistance against calcineurin inhibitors. We also found that the level of expression of *bycA* was significantly higher in the wild-type strain treated with FK506 and in the *cnbR*Δ mutants but was significantly lower in the wild-type strain without FK506 treatment. These findings suggest that *bycA* is a negative regulator of hyphal growth and/or a positive regulator of yeast growth in *Mucor* and that calcineurin suppresses expression of the *bycA* gene at the mRNA level to promote hyphal growth. BycA is involved in the *Mucor* hypha-yeast transition as our data demonstrate positive correlations among *bycA* expression, protein kinase A activity, and *Mucor* yeast growth. Also, calcineurin, independently of its role in morphogenesis, contributes to virulence traits, including phagosome maturation blockade, host cell damages, and proangiogenic growth factor induction during interactions with hosts.

## Abstract (precis)

*Mucor* is intrinsically resistant to most known antifungals, which makes mucormycosis treatment challenging. Calcineurin is a serine/threonine phosphatase that is widely conserved across eukaryotes. When calcineurin function is inhibited in *Mucor*, growth shifts to a less virulent yeast growth form, which makes calcineurin an attractive target for development of new antifungal drugs. Previously, we identified two distinct mechanisms through which *Mucor* can become resistant to calcineurin inhibitors involving Mendelian mutations in the gene for FKBP12, including mechanisms corresponding to calcineurin A or B subunits and epimutations silencing the FKBP12 gene. Here, we identified a third novel mechanism where loss-of-function mutations in the amino acid permease corresponding to the *bycA* gene contribute to resistance against calcineurin inhibitors. When calcineurin activity is absent, BycA can activate protein kinase A (PKA) to promote yeast growth via a cAMP-independent pathway. Our data also show that calcineurin activity contributes to host-pathogen interactions primarily in the pathogenesis of *Mucor.*

## INTRODUCTION

Mucormycosis is a severe life-threatening infection caused by fungi belonging to the order Mucorales (1). People with weakened immune systems due to diabetes mellitus, neutropenia, hematological disorders, and solid-organ transplantation are at the highest risk of acquiring this infection (2, 3). It is the third most common invasive fungal infection in hematological and allogeneic stem transplantation patients following candidiasis and aspergillosis (1, 4). Over the past decades, there has been a global and ongoing rise in the incidence of mucormycosis, primarily due to the increasing number of diabetic patients and increased use of immunosuppressive drugs (5–10). Mucormycosis is also on the rise in immunocompetent individuals (11–14). Mucorales species grow as molds in the environment and produce sporangiospores that can enter the host via inhalation resulting in pulmonary infection, through the skin due to trauma resulting in cutaneous infections, or through the nasal passages resulting in rhinocerebral infections (15–17). The spores can disseminate within the host, resulting in 95% to 100% mortality even with antifungal drug treatment (18). Mucorales species are intrinsically resistant to most antifungals; thus, Mucorales infections are very difficult to treat, and surgery is often required (19).

Calcineurin is a calcium-calmodulin-dependent phosphatase conserved widely across eukaryotes, including pathogenic fungi (20, 21). Calcineurin is a heterodimer consisting of a catalytic subunit and a regulatory subunit, and both subunits are required for calcineurin function. The role of calcineurin varies depending on the fungal species; for example, calcineurin is required for growth at high temperature (37°C) and at alkaline pH in Cryptococcus neoformans and Cryptococcus gattii (22–24), while calcineurin contributes to azole tolerance and is required for survival in serum, among other functions, in *Candida* spp. (20, 25). In Aspergillus fumigatus, calcineurin mutants exhibit delayed germination, hyphal growth with irregular branching, and abnormal septa (26). We have previously shown that calcineurin regulates dimorphism in *Mucor* spp., where the calcineurin inhibitor FK506 (tacrolimus) forces *Mucor* to grow only as yeast (27).

Hyphal morphology is the predominant growth mode for *Mucor* spp.; however, by modulation of respiratory conditions, *Mucor* can be forced to grow as yeast as well (28). While aerobic conditions promote hyphal growth, conditions that include low levels of oxygen and high levels of carbon dioxide enforce yeast growth (29–32). Targeting components involved in mitochondrial or lipid metabolism can also promote yeast growth, even under aerobic conditions (33–36). In addition, previous studies have shown that the addition of cyclic AMP to *Mucor* in culture results in activation of cAMP-dependent kinase protein kinase A (PKA) and promotes yeast growth (37–40). Wolff et al. also showed that higher levels of expression of PKA regulatory and catalytic subunits are exhibited during anaerobic yeast growth than during aerobic hyphal growth in *Mucor* (41). *Mucor* spp. have four isoforms of PKA regulatory subunits, and each is differentially expressed depending on the growth conditions (38, 39). Calcineurin is involved in the genetic regulation of *Mucor* dimorphism, as deletion of the gene encoding the regulatory subunit of calcineurin (CnbR) resulted in yeast-locked growth, even under aerobic conditions (27). *cnbR*Δ mutants were previously shown to be avirulent in a wax moth host model (27), thereby indicating that calcineurin is an attractive target for antifungal treatment in mucormycosis. Also, mucormycosis incidence is low in patients receiving FK506 as an immunosuppressant (42). *cnbR*Δ mutants are also more susceptible to antifungal drugs such as amphotericin B (Ambisome), micafungin, and posaconazole (43).

The cellular receptor for calcineurin is FKBP12, a member of the immunophilin protein family with *cis*-*trans* peptide prolyl isomerase activity (44). When FK506 is bound to FKBP12, it inhibits calcineurin phosphatase activity by binding to the calcineurin interface between the catalytic A subunit and the regulatory B subunit, thereby preventing access of substrates to the active site (45, 46). FKBP12 also binds to rapamycin to inhibit the Tor pathway (47), and mutations in the FKBP12 gene confer resistance to both FK506 and rapamycin. Amino acid substitutions in the calcineurin regulatory B and catalytic A subunit surfaces that interact with the FKBP12-FK506 complex can also result in resistance to FK506 (48, 49). Another immunophilin, cyclophilin A (Cyp), serves as a cellular receptor for the drug cyclosporine (CsA). When bound to Cyp, CsA inhibits calcineurin in a manner similar to that seen with FKBP12-FK506 (50). Disruption of the gene encoding Cyp therefore confers resistance to CsA.

In our previous studies, calcineurin inhibitor-resistant *Mucor* strains, which exhibit hyphal growth instead of yeast growth, were found to have mutations in the FKBP12 gene or the calcineurin catalytic A gene or regulatory B subunit gene (27, 43, 51, 52). In addition, Calo et al. found that *Mucor* can also silence the FKBP12 gene to become transiently resistant to FK506 and rapamycin via an RNA interference (RNAi)-dependent epimutation pathway (51, 52). In this study, we isolated mutants that do not employ the known calcineurin inhibitor resistance mechanisms. We identified a novel mechanism through which *Mucor* can become resistant to calcineurin inhibitors. We found that mutations or deletions in a novel gene, *bycA* (bypass of calcineurin), encoding an amino acid permease that confers resistance to calcineurin inhibitors or loss of calcineurin regulatory B subunit. This gene has not been previously described as involved in the calcineurin pathway, morphogenesis, or virulence in Mucorales. As a result, *bycA* mutation allowed us to separate the yeast-hypha morphology switch from calcineurin function and to demonstrate that calcineurin, independently of its function in regulating morphology, contributes to *Mucor*-host interactions.

## RESULTS

### Isolation of calcineurin bypass mutants in the yeast-locked *cnbR*Δ mutant background.

**FIG 1:**
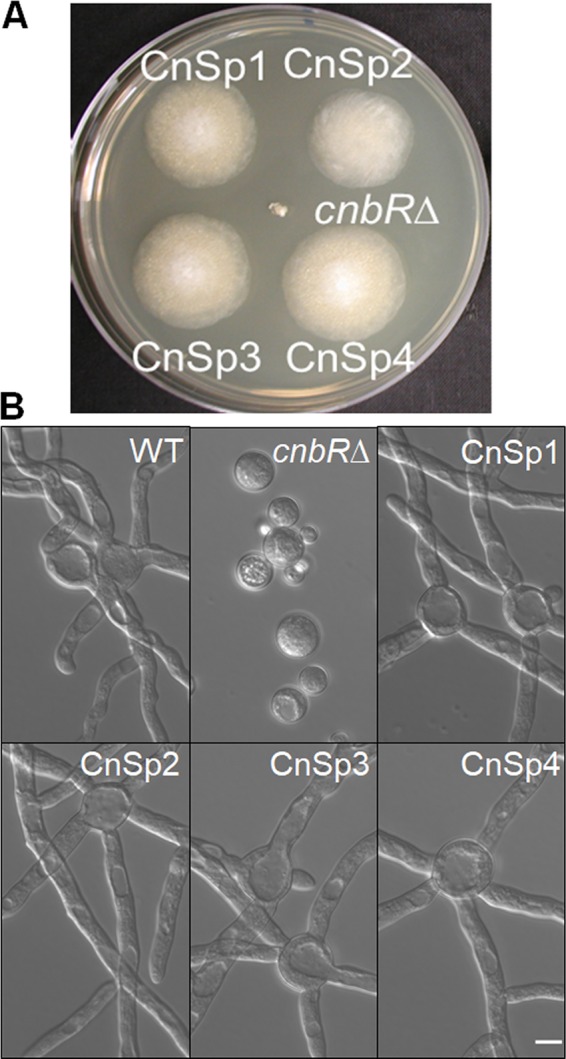
Calcineurin suppressor (CnSp) mutations in the *cnbR*Δ mutant background restore hyphal growth. (A) Growth of calcineurin suppressor mutants (CnSp1 to CnSp4) and *cnbR*Δ mutant on a YPD agar plate at 30°C for 4 days postinoculation. While the *cnbR*Δ mutant shows smaller yeast colonies, the CnSp mutants show larger hyphal colonies. (B) The *Mucor* WT (R7B), *cnbR*Δ mutant, or CnSp mutants were grown overnight in liquid YPD medium with shaking at 30°C. Micrographs show that the CnSp mutants exhibit hyphal growth like that seen with the WT (scale bar = 10 μm).

The *cnbR*Δ mutant grows exclusively as a yeast (27). However, we isolated spontaneous mutants that exhibit hyphal growth in the *cnbR*Δ background. When the mutant (10^3^ cells per spot) was grown in solid yeast extract-peptone-dextrose (YPD) medium at 30°C for a prolonged incubation (exceeding 5 days), we observed that hyphal sectors emerged from the yeast colonies (see Fig. S1A in the supplemental material). After two rounds of single-streak dilutions, 17 independently isolated mutants (calcineurin suppressor [CnSp] mutants) in the *cnbR*Δ background were isolated (Fig. 1) (Table 1; see also Fig. S1B). The hyphae of the mutants continued to grow, producing aerial hyphae decorated with sporangiophores containing sporangiospores (asexual spores [referred to here as “spores”]). This finding shows that these mutants can complete the entire vegetative cycle without a functional calcineurin. We previously demonstrated that calcineurin function is required for hyphal growth (27). However, these spontaneous mutants do not have a functional calcineurin but exhibit filamentation and produce spores. Thus, the mutants carry a genetic suppressor mutation(s) of the *cnbR*Δ mutation.

**TABLE 1:** Characterization of the bypass mutants isolated in the *cnbR*Δ mutant background

Strain	Genotype in the *bycA* allele	Remark	Type of mutation
CnSp1	1350G→A		Nonsense
CnSp2	Long deletion around *bycA*	Whole genome sequenced	
CnSp3	2030C→A	Whole genome sequenced	Nonsense
CnSp4	361_1579del	Whole genome sequenced	Frameshift
CnSp5	Potential deletion	No *bycA* PCR product	
CnSp6	No mutation in *bycA* allele		
CnSp7	995C→T	Whole genome sequenced	Missense
CnSp8	1921_1922insTGACATTGCTTCAGCAG	Whole genome sequenced	Frameshift
CnSp9	320T→C		Missense
CnSp10	2319_2320insC	Whole genome sequenced	Frameshift
CnSp11	2129_2130insTCACC		Frameshift
CnSp12	Potential deletion	No *bycA* PCR product	
CnSp13	1784_1793delTCAATTTCAT		Frameshift
CnSp14	566T→A		Nonsense
CnSp15	2238_2239insTC		Frameshift
CnSp16	No mutation in *bycA* allele		
CnSp17	1732C→T		Nonsense

10.1128/mBio.02949-19.1 FIG S1 Emergence of hyphal sectors from *cnbR*Δ mutants and isolation of calcineurin suppressor mutants. (A) *cnbR*Δ mutants were plated on YPD agar for 5 or more days at 30°C. (B) The hyphae emerging from the yeast colonies were transferred to fresh YPD agar for further propagation. (C) Calcineurin suppressor (CnSP) mutants produced hyphae decorated with sporangiophores containing sporangiospores like the WT results (scale bar = 20 μm). Download FIG S1, TIF file, 2.4 MB. Copyright © 2020 Vellanki et al. 2020 Vellanki et al. This content is distributed under the terms of the Creative Commons Attribution 4.0 International license.

### Isolation of calcineurin bypass mutants in the *cnaB*Δ background.

**FIG 2:**
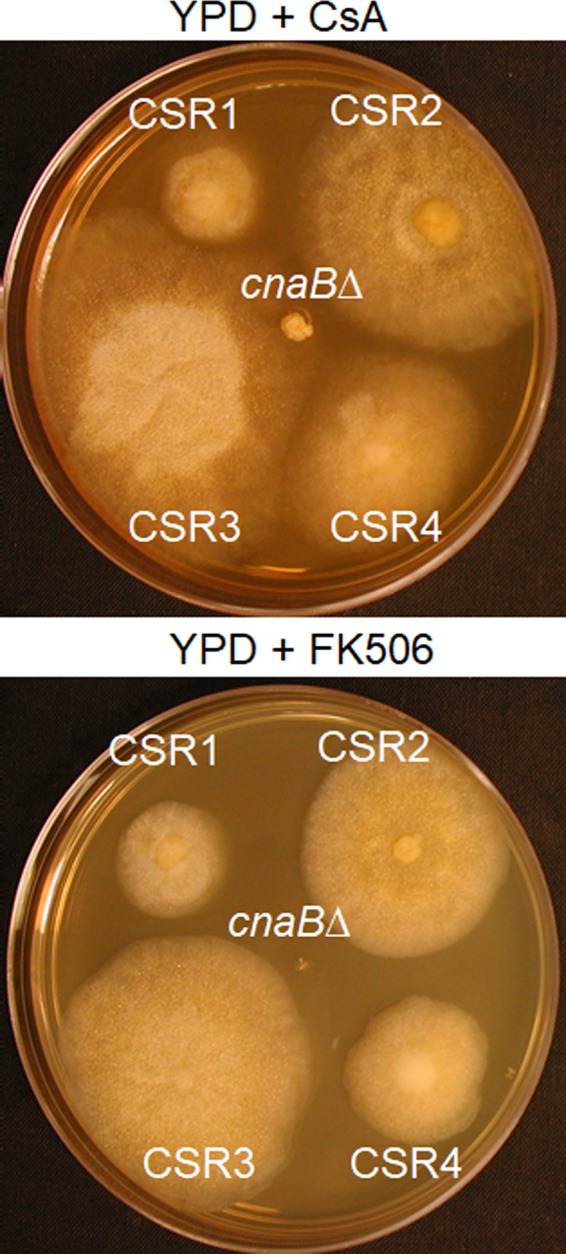
Calcineurin suppressor mutations in the *cnaB*Δ mutant background confer resistance to calcineurin inhibitors. The images show growth of cyclosporine-resistant mutants (CSR) exhibiting hyphal growth and of *cnaB*Δ mutants exhibiting yeast growth under conditions of incubation on YPD agar with CsA (100 μg/ml) (top panel) or FK506 (1 μg/ml) (bottom panel) for 4 days at 30°C. CSR1, CSR2, CSR3, and CSR4 are shown. The other CSR mutants exhibited a similar CsA resistance phenotype (data not shown).

The CsA calcineurin inhibitor does not fully force *Mucor* to grow as yeast, unlike FK506. Instead, CsA fully inhibits hyphal growth and enforces a yeast-locked phenotype only when *Mucor* lacks the *cnaB* gene, which is one of the three calcineurin catalytic subunit genes (*cnaA*, *cnaB*, and *cnaC*) (43). We grew the *cnaB*Δ mutant on YPD medium containing 2 μg/ml of CsA, and the mutant grew exclusively as yeast because calcineurin was inhibited by CsA. Similarly, we observed that hyphal sectors emerged from yeast colonies as the strains became resistant to CsA after prolonged incubation on CsA medium (Fig. S2). After performing two rounds of single-streak dilutions, we isolated 19 independently derived CsA-resistant (CSR) mutants (Table 2). The mutants exhibited hyphal growth in the presence of either CsA or FK506 (Fig. 2).

**TABLE 2:** Characterization of the calcineurin bypass mutants isolated in the *cnaB*Δ mutant background

Strain	Genotype in the *bycA* allele	Remark	Type of mutation
CSR1	C1240del		Frameshift
CSR2	Potential deletion	No *bycA* PCR product	
CSR3	733 C→A		Missense
CSR4	633delG	Whole genome sequenced	Frameshift
CSR5	407C→T	Whole genome sequenced	Missense
CSR6	2188delC	Whole genome sequenced	Frameshift
CSR7	806_825delCCTACCCGCCAGTGGAAGCA		Frameshift
CSR8	1711delG		Frameshift
CSR9	2188delC	Whole genome sequenced	Frameshift
CSR10	1649delT	Whole genome sequenced	Frameshift
CSR11	578G→A		Nonsense
CSR12	621delT		Frameshift
CSR13	2296_2297insAAT		Frameshift
CSR14	218G→A		Nonsense
CSR16	2263delC	Whole genome sequenced	Frameshift
CSR28	215G→A		Missense
CSR29	No mutation in *bycA* allele		
CSR35	No mutation in *bycA* allele		
CSR38	No mutation in *bycA* allele		

10.1128/mBio.02949-19.2 FIG S2 Emergence of hyphal sectors from the *cnaB*Δ mutant grown on CsA-containing medium. *cnaB*Δ mutants were grown in the presence of CsA (2 μg/ml) for 5 or more days at 30°C. This resulted in the emergence of hyphal sectors from yeast colonies. Download FIG S2, TIF file, 1.2 MB. Copyright © 2020 Vellanki et al. 2020 Vellanki et al. This content is distributed under the terms of the Creative Commons Attribution 4.0 International license.

Surprisingly, none of the mutants carried Mendelian mutations in genes for the calcineurin A subunit or B subunit, FKBP12, or two subtypes of cyclophilin A (*cypA* and *cypB*) (data not shown). In addition, the mutants did not carry epimutations in the *cypA* and *cypB* genes, and small RNA blots did not reveal any small RNAs derived from the *cypA* and *cypB* genes (51) (data not shown). Therefore, we hypothesized that the CSR mutants were also calcineurin bypass mutants that do not require calcineurin activity for hyphal growth.

### Spontaneous mutations in the *bycA* gene result in phenotypes of suppression of the calcineurin mutation.

To characterize the mutation(s) that results in bypass of the calcineurin requirement, we sequenced the whole genomes of 6 CnSp and 6 CSR mutant strains, along with wild-type (WT) strain MU402, which was used for transformation to obtain the *cnbR* and *cnaB* mutants with an Illumina HiSeq platform. The whole genomes of each mutant strain were compared to that of the wild-type MU402 strain. Surprisingly, all 12 mutants carried DNA sequence modifications in a single common locus (Table 1 and Table 2). The modifications included an insertion of short sequences, single nucleotide polymorphisms, or deletions of a shorter or longer region in the locus. The gene in the locus was designated *bycA* (bypass of calcineurin). We further sequenced the *bycA* gene in the remaining CSR and CnSp mutants and found mutations in the *bycA* gene in all but 5 mutants (Table 1 and Table 2). Of the 36 suppressor mutants, 31 contained mutations in the *bycA* gene that could result in suppression of the calcineurin mutation.

### *bycA*Δ mutants are resistant to calcineurin inhibitors, and *bycA*Δ *cnbR*Δ double mutants exhibit a hyphal morphology.

**FIG 3:**
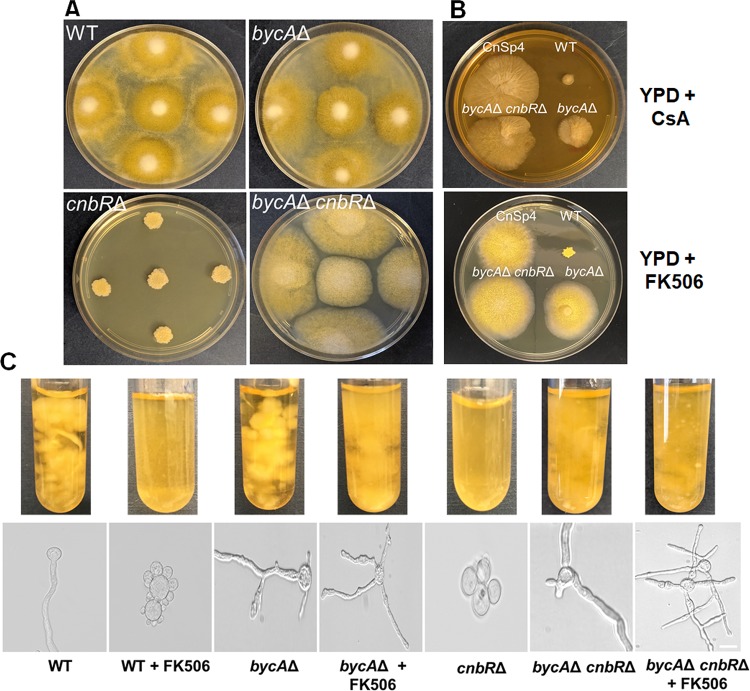
The *bycA*Δ single mutant and *bycA*Δ *cnbR*Δ double mutant are resistant to calcineurin inhibitors. (A) After 3 days of growth on solid YPD agar, the *cnbR*Δ mutant grew as yeast; however, despite no calcineurin function, the *bycA*Δ *cnbR*Δ double mutant exhibited hyphal growth like that shown by the WT and the *bycA*Δ mutant. (B) In the presence of CsA (upper panel; 100 μg/ml), the *bycA*Δ *cnbR*Δ double mutant and CnSp4 formed hyphal colonies that were larger than those formed by the WT, indicating resistance to CsA. No major difference in colony size was noted between the *bycA*Δ mutant and the WT. In the presence of FK506 (lower panel; 1 μg/ml), the WT formed a smaller yeast colony whereas the *bycA*Δ mutant, *bycA*Δ *cnbR*Δ double mutant, and CnSp4 each formed a larger hyphal colony. (C) When *Mucor* was grown overnight in YPD medium containing FK506 (1 μg/ml) at 30°C with shaking, the *bycA*Δ mutant, *bycA*Δ *cnbR*Δ double mutant, and CnSp4 mutant exhibited resistance to FK506 as evidenced by larger biomass and hyphal morphology, whereas the WT cells were sensitive to FK506 as they not only formed less biomass but also grew as yeast. As expected, the *cnbR*Δ mutant remained in its yeast-locked form (scale bar = 20 μm).

**FIG 4:**
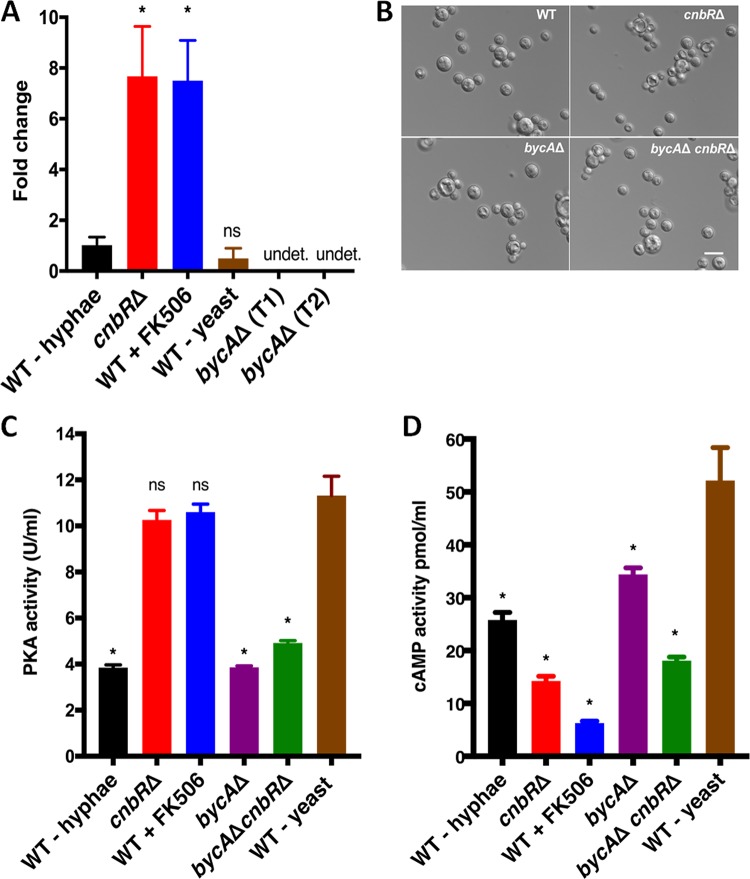
BycA is involved in the *Mucor* hypha-yeast transition under aerobic conditions. (A) Reverse transcriptase quantitative PCR showed that in the absence of calcineurin function (*cnbR*Δ and WT + FK506; yeast morphology), *bycA* expression was 6-fold higher than the level seen with the WT (hyphal morphology) with calcineurin function, suggesting that calcineurin regulates *bycA* expression at the mRNA level. When the *Mucor* WT was grown anaerobically under high-CO_2_ conditions (see Materials and Methods), it grew as yeast; however, there was no significant difference in *bycA* expression compared to the level seen with the WT-hypha group. One-way analysis of variance (ANOVA) data were significant (*P* = 0.0001). Dunnett’s *post hoc* test was used to compare *cnbR*Δ, WT + FK506, WT-yeast results with those seen with the WT-hypha group (*, *P* < 0.05; ns, not statistically significant; undet., undetected). As expected, no expression of the *bycA* gene was detected in either of the *bycA*Δ mutants. (B) When WT, *cnbR*Δ, *bycA*Δ, and *bycA*Δ *cnbR*Δ mutants were grown anaerobically overnight under high-CO_2_ conditions, they all exhibited a yeast morphology, thereby suggesting that neither calcineurin nor BycA has a role in anaerobic morphological pathways (scale bar = 20 μm). (C) Crude protein extracts (0.5 μg) were used to measure overall PKA activity. The WT-hypha and the *bycA*Δ mutant showed significantly lower PKA activity than WT-yeast (grown anaerobically). In WT + FK506 and the *cnbR*Δ mutant, PKA activity remained higher; however, the *bycA*Δ *cnbR*Δ mutant showed lower PKA activity despite possessing no calcineurin function. One-way ANOVA data were significant (*P* < 0.0001). Dunnett’s multiple-comparison test was used to compare each group with WT-yeast (*, *P* < 0.05). (D) Crude extracts from 60 mg biomass were used to measure overall cAMP activity. Yhe WT + FK506 and *cnbR*Δ strains showed significantly lower cAMP activity than WT-yeast, thereby suggesting that under aerobic conditions, PKA activity is elevated in *Mucor* yeast in a cAMP-independent manner. One-way ANOVA data were significant (*P* < 0.0001). Dunnett’s multiple-comparison test was used to compare each group with WT-yeast (*, *P* < 0.05).

We further verified that *bycA* is associated with the calcineurin pathway by generating *bycA* deletion mutants. The *bycA* gene in wild-type strain MU402 was replaced with the *pyrG-dpl237* marker (53), and gene replacement by recombination was confirmed by 5′ and 3′ junction PCR (Fig. S3), open reading frame (ORF)-spanning PCR (Fig. S3), and Southern blotting (not shown). We also performed reverse transcriptase PCR (RT-PCR) to confirm the *bycA* gene is not expressed in the mutants (see Fig. 4A). Two independent mutants, MSL47.1 and MSL47.2 (*bycA*Δ::*pyrG-dpl237*), were obtained. The *bycA*Δ mutants exhibited hyphal growth and showed no growth defects (Fig. 3A). On solid agar medium containing FK506, a *bycA*Δ mutant and CnSp4 produce a larger hyphal colony and aerial hyphae, whereas the wild-type strain formed a smaller yeast colony (Fig. 3B). These results indicate that *bycA*Δ mutants are resistant to FK506.

10.1128/mBio.02949-19.3 FIG S3 Confirmation of disruption of the *bycA* gene by junction PCR and ORF-spanning PCR. (A) Illustration of the *bycA*Δ::*pyrG*-*dpl237* and *bycA* alleles with ∼1-kb upstream and downstream flanking sequence. P1 (SL3) and P4 (SL8) recognize sequences outside the disruption cassette. P2 (SCL566) and P3 (SCL567) recognize *pyrG*-*dpl237*. P5 (SL182) and P6 (SL183) recognize *bycA*. (B) At the 5′ end, P1 and P2 amplified a 2,203-bp region, and at the 3′ end, P3 and P4 amplified a 1,994-bp region in *bycA*Δ::*pyrG*-*dpl237*. The primer pairs did not produce a fragment in the WT. P5 and P6 amplified a 690-bp region within the *bycA* gene, so no amplification was noted in the mutants. Images are not to scale. Download FIG S3, TIF file, 0.4 MB. Copyright © 2020 Vellanki et al. 2020 Vellanki et al. This content is distributed under the terms of the Creative Commons Attribution 4.0 International license.

Under conditions of growth on solid YPD media containing CsA (50 μg/ml), interestingly, the *bycA*Δ mutants were not fully resistant to CsA and instead exhibited colony size comparable to that seen with the wild type (R7B). However, CnSp4 exhibited larger colonies on media containing CsA (Fig. 3B). It is possible that the CnaB catalytic A subunit is partially resistant to the inhibition by CsA, during which Cyp-CsA may not interfere with the function of CnaB-CnbR-calmodulin while still inhibiting CnaA-CnbR-calmodulin and CnaC-CnbR-calmodulin. The *bycA*Δ mutants still harbor an intact *cnaB* gene, and it is possible that partial inhibition of CnaB could result in a calcineurin-independent phenotype. Therefore, *bycA*Δ mutants in the *cnaB*Δ background exhibited full resistance against CsA, representing a result which has yet to be elucidated.

We further disrupted the *cnbR* gene in the *bycA*Δ background to generate *bycA*Δ *cnbR*Δ double mutants. The deletion and the absence of expression of *cnbR* were confirmed by PCR and RT-PCR (Fig. S4 and Fig. S5), respectively, and by Southern blotting (data not shown). Two independent mutants, MSL68.1 and MSL68.2 (*bycA*Δ::*pyrG-dpl237 cnbR*Δ::*leuA*), were generated. As shown in Fig. 3A, while the *cnbR*Δ mutants grow exclusively as yeast, *bycA*Δ *cnbR*Δ double mutants exhibited filamentous growth like that exhibited by the wild-type strain and the *bycA*Δ mutant. *bycA*Δ *cnbR*Δ mutants are also completely resistant to either FK506 or CsA (Fig. 3B), as they exhibit normal hyphal growth even in the presence of calcineurin inhibitors (Fig. 3B and C). Taken together, our results genetically validate our hypothesis that *bycA* mutations bypass the requirement of calcineurin for hyphal growth or suppress the lack of calcineurin.

10.1128/mBio.02949-19.4 FIG S4 Confirmation of disruption of the *cnbR* gene in the *bycA*Δ background by junction PCR and ORF-spanning PCR. (A) Illustration of the *cnbR*Δ::*leuA* deletion in the *bycA*Δ::*pyrG*-*dpl237* strain and *cnbR* alleles with ∼1-kb upstream and downstream flanking sequence. P1 (SL243) and P4 (SL244) recognize sequences outside the disruption cassette. P2 (SL391) and P3 (SL392) are specific for *leuA*. P5 (SCL578) and P6 (SCL579) are specific for *cnbR*. (B) At the 5′ end, P1 and P2 amplified a 2,388-bp region, and at the 3′ end, P3 and P4 amplified a 1,620-bp region in the *cnbR*Δ::*leuA* region. The primer pair did not produce a fragment in the WT. P1 and P4 produced a 5,711-bp product in the mutant and a 3,342-bp product in the WT. P5 and P6 amplified a 170-bp region within the *cnbR* gene, so no amplification is noted in the mutants. Image is not to scale. Download FIG S4, TIF file, 0.7 MB. Copyright © 2020 Vellanki et al. 2020 Vellanki et al. This content is distributed under the terms of the Creative Commons Attribution 4.0 International license.

10.1128/mBio.02949-19.5 FIG S5 Confirmation of disruption of the *cnbR* gene in the *bycA*Δ background by RT-qPCR. Primers SCL578 and SCL579 were used to perform qPCR on the cDNA obtained from the WT and mutants (see Materials and Methods). Actin (SCL368 and SCL369) served as a control. No *cnbR* expression was detected in the *bycA*Δ *cnbR*Δ double mutant. Download FIG S5, TIF file, 0.1 MB. Copyright © 2020 Vellanki et al. 2020 Vellanki et al. This content is distributed under the terms of the Creative Commons Attribution 4.0 International license.

### Calcineurin regulates BycA at the mRNA level.

To determine a genetic link between calcineurin and BycA, the expression of *bycA* was examined under conditions in which calcineurin was either functional or nonfunctional. As shown in Fig. 4A, *bycA* gene expression was significantly higher than the WT level under conditions in which calcineurin was not functional such as in the *cnbR*Δ mutant or in the wild type in the presence of FK506. These results suggest that calcineurin negatively regulates the *bycA* gene at the mRNA level and that BycA expression is positively correlated with *Mucor* yeast growth.

Under anaerobic (or microaerobic) conditions with high levels of CO_2_, *Mucor* also grows as a yeast (54). Interestingly, under those conditions, *bycA* expression remained low (Fig. 4A). When *bycA*Δ or *bycA*Δ *cnbR*Δ mutants were grown anaerobically with high CO_2_ levels, they still grew as yeast (Fig. 4B). These results suggest that BycA is involved in aerobic yeast growth but not in anaerobic yeast growth. Whether calcineurin inhibits the expression of the *bycA* gene transcriptionally or regulates the stability of the *bycA* mRNA remains to be elucidated.

### BycA serves as a link between calcineurin and protein kinase A.

Previous studies by our groups and others have shown that increased levels of bicarbonate ions through addition to *Mucor* culture or as a consequence of growth under high-carbon-dioxide conditions are sufficient to induce yeast growth (27, 31), as the bicarbonate ions can activate adenylyl cyclase, resulting in the generation of cAMP and, in turn, in the activation of cAMP-dependent kinase, namely, protein kinase A (PKA) (55, 56) (see Fig. 8). Studies have also shown that there is an inverse relationship between PKA and hyphal morphology as the PKA regulatory subunit gene that inhibits the activity of PKA is more highly expressed during hyphal growth (41). Also, high levels of cAMP promote yeast growth, while low levels of cAMP are linked with hyphal morphology (28). Interestingly, even under aerobic conditions when *Mucor* was forced to grow as yeast by inhibiting calcineurin activity either genetically or by using FK506, the overall cellular PKA activity remained high (27). Therefore, PKA plays pivotal roles in yeast growth. Interestingly, in the wild type when calcineurin is fully functional, the cellular PKA activity is low (27), suggesting an inverse correlation between PKA and calcineurin levels during morphogenesis. However, it was not clear how calcineurin and PKA are linked.

BycA is a putative amino acid permease and is predicted to have 10 transmembrane domains and 1 pectinesterase domain (Fig. S6). As shown in Table 3, when *cnbR*Δ and *bycA*Δ *cnbR*Δ mutants were grown on YNB medium with methionine or threonine or arginine as a sole nitrogen source, the *cnbR*Δ mutants exhibited growth levels similar to those seen on YNB complete medium, while *bycA*Δ *cnbR*Δ mutants did not produce hyphal mass. This shows that BycA is a bona fide amino acid permease. In Saccharomyces cerevisiae and Candida albicans, the general amino acid permease Gap1 is known to activate PKA in a cAMP-independent manner (57, 58). We hypothesized that in the absence of calcineurin activity, BycA may activate PKA to promote yeast growth. To this end, we measured overall cellular PKA activity in the presence or absence of the *bycA* gene. As shown in Fig. 4C, PKA activity was significantly higher in WT-yeast, in the *cnbR*Δ mutant, and in the WT strain subjected to FK506 treatment (WT + FK506) (when *bycA* expression was significantly higher) than in the WT hyphae (when *bycA* expression is low). On the other hand, the *bycA*Δ or *bycA*Δ *cnbR*Δ mutants exhibited significantly lower cellular PKA activity when the *bycA* gene was deleted than were seen under the conditions in which the *bycA* gene was expressed. No significant differences were observed between the WT hyphae and the *bycA*Δ or *bycA*Δ *cnbR*Δ isolates.

**TABLE 3:** Summary of *cnbR*Δ single mutant and *bycA*Δ *cnbR*Δ double mutant growth in the presence of methionine, threonine, and arginine as sole nitrogen source

Nitrogen source	Growth level^*a*^	
	*cnbR*Δ mutant	*bycA*Δ *cnbR*Δ mutant
None	−	−
Methionine	+	−
Threonine	+	−
Arginine	+	−
Casamino Acids	++	++

a −, no growth; +, low growth level; ++, high growth level.

10.1128/mBio.02949-19.6 FIG S6 Predicted structure of BycA showing 10 transmembrane domains and 1 cytosolic domain. BycA is predicted to have 10 transmembrane domains and 1 cytosolic domain. The transmembrane domains were predicted with the TMHMM 2.0 software package (http://www.cbs.dtu.dk/services/TMHMM/). The predicted cytosolic region contains a pectinesterase domain. Download FIG S6, TIF file, 0.2 MB. Copyright © 2020 Vellanki et al. 2020 Vellanki et al. This content is distributed under the terms of the Creative Commons Attribution 4.0 International license.

Interestingly, measurements of cAMP levels showed that the cAMP levels were significantly lower in the *cnbR*Δ and WT + FK506 yeast groups than in the WT-yeast group despite high PKA activity (Fig. 4D). This suggests that under aerobic conditions when calcineurin is absent, BycA increases PKA activity via a cAMP-independent pathway, a result which has yet to be elucidated.

### Phagosome maturation blockade upon phagocytosis by macrophages is dependent on calcineurin rather than morphology.

**FIG 5:**
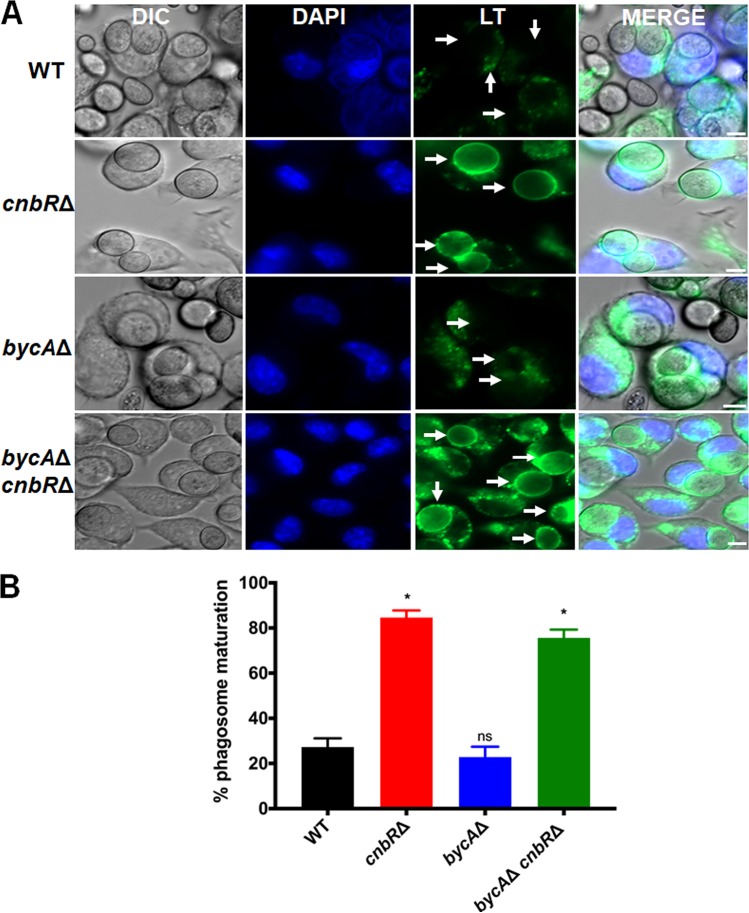
Phagosome maturation in macrophages containing *Mucor* is dependent on pathogen calcineurin function and not morphology. (A) J774.A1 macrophages (5 × 10^5^) were challenged with *Mucor* spores or yeast at an MOI of 1 along with LysoTracker Green DND-26 and Hoechst 33342 stain (blue). White arrows indicate where phagosome maturation should be observed in the field (scale bar = 5 μm). DIC, differential interference contrast; DAPI, 4′,6-diamidino-2-phenylindole; LT, LysoTracker. (B) The macrophages containing the *cnbR*Δ single mutant and the *bycA*Δ *cnbR*Δ double mutant underwent significantly higher phagosome maturation than those containing the WT. Data are shown as percent maturation. The numbers (*n*) of macrophages containing *Mucor* counted for each group were as follows: WT = 480; *bycA*Δ mutant = 290; *cnbR*Δ mutant = 279; *bycA*Δ *cnbR*Δ mutant = 413. One-way ANOVA data were significant (*P* < 0.0001). Dunnett’s multiple-comparison test was used to compare each group to the WT (*, *P* < 0.05).

Phagocytosis followed by phagosome maturation or acidification in macrophages is an essential innate immune pathway to control pathogens. We have previously shown that when the macrophage cell line J774.A1 and primary macrophages were challenged with wild-type spores or *cnbR*Δ yeast, they rapidly phagocytosed both spores and yeast; however, only the macrophages with *cnbR*Δ yeast underwent phagosome maturation (43). This indicates that *Mucor* spores escape innate immunity by blocking phagosome maturation as spores survive better than *cnbR*Δ yeast during coculture with macrophages (Fig. S9). However, it was not clear if the blockade of phagosome maturation by *Mucor* is dependent on its morphology or on the presence of functional calcineurin because yeast cells lack calcineurin function. To address this issue, we cocultured WT spores, *cnbR*Δ yeast, and *bycA*Δ *cnbR*Δ double mutant spores with macrophages to monitor phagosome maturation. Macrophages containing *Mucor* spores or yeast cells were stained with Lysotracker Green DND-26 (Thermofisher). Lysotracker stains only acidic organelles in cells such as lysosomes or mature phagosomes and therefore can be used to determine whether phagosomes containing *Mucor* cells are acidic, an indication of phagosome maturation. As shown in Fig. 5, only about 20% of macrophages challenged with either wild-type (*n* = 480) or *bycA*Δ (*n* = 290) spores underwent phagosomal maturation, which is significantly lower than the proportion seen with macrophages challenged with the *cnbR*Δ mutant (*n* = 279; yeast-locked). Interestingly, ∼80% of phagosomes containing *bycA*Δ *cnbR*Δ double mutants (*n* = 413) spores underwent maturation. Our data suggest that a novel downstream function of calcineurin, independent of its function in governing morphology, is involved in the inhibition of phagosome maturation.

### *Mucor* without functional calcineurin causes reduced cell damage and induces lower levels of FGF-2 expression in endothelial cells.

**FIG 6:**
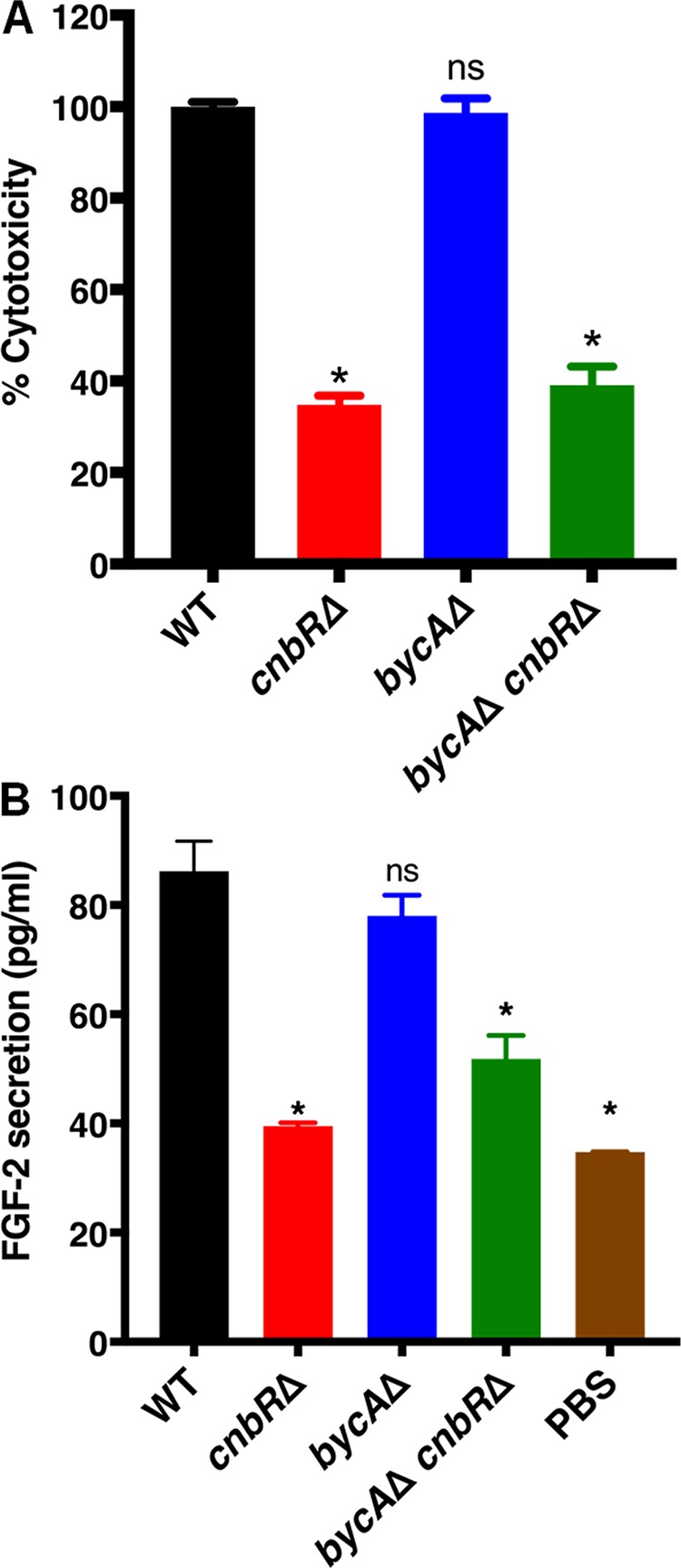
Calcineurin mutants cause less endothelial cell damage and FGF-2 protein secretion. A total of 5 × 10^3^ HUVECs were challenged with *Mucor* spores or yeast at an MOI of 10. After 24 h, the supernatant was collected. (A) Levels of LDH (indicating cytotoxicity) were quantified. Calcineurin mutants caused less damage than the WT. One-way ANOVA data were significant (*P* < 0.0001). Dunnett’s multiple-comparison test was used to compare each group to the WT (*, *P* < 0.05). (B) FGF-2 levels were quantified using ELISA. The WT strain induced significantly higher FGF-2 protein secretion than the calcineurin mutants. One-way ANOVA data were significant (*P* < 0.0001). Dunnett’s multiple-comparison test was used to compare each group to the WT (*, *P* < 0.05).

Mucormycosis is an angioinvasive disease, and hence Mucorales interaction and the subsequent damage to the endothelium lining the blood vessels are important steps in disease pathology (59). We have previously shown that *Mucor* hyphae but not yeast cells (*cnbR*Δ mutant) induce fibroblast growth factor-2 (FGF-2) protein expression in lymphoblastoid cell lines and bone marrow macrophages (43, 60). We wanted to determine the extent to which calcineurin contributes to the endothelial damage and FGF-2 protein response by *Mucor.* To this end, we used human umbilical vein endothelial cells (HUVECs) and challenged cells with the wild type, the *bycA*Δ single mutant, the *cnbR*Δ single mutant, or the *bycA*Δ *cnbR*Δ double mutant for a period of 24 h. HUVECs challenged with the *cnbR*Δ single mutant and the *bycA*Δ *cnbR*Δ double mutant showed 70% less cytotoxicity than HUVECs challenged with wild-type or *bycA*Δ spores as quantified by measuring lactate dehydrogenase (LDH) levels (Fig. 6A) and significantly less FGF-2 protein production (Fig. 6B). These data suggest a morphology-independent function of calcineurin in regulating *Mucor* interaction with endothelial cells.

### *bycA*Δ *cnbR*Δ double mutants are less virulent in a Galleria mellonella host model of mucormycosis.

**FIG 7:**
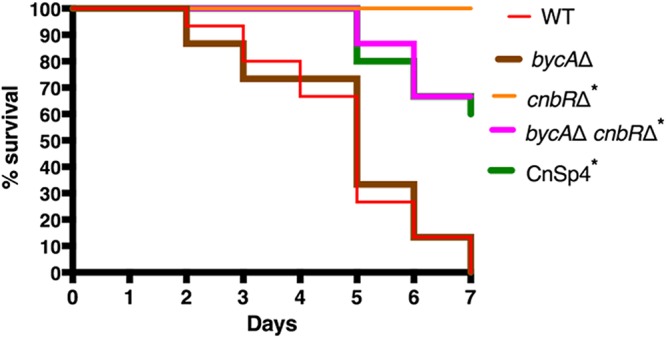
Calcineurin mutants were less virulent in a Galleria mellonella (wax moth) model of mucormycosis. Wax moth larvae (*n* = 15/group) were inoculated with 1 × 10^4^ *Mucor* spores or yeast in a mixture containing 2 μl PBS via injection into the last left proleg and were monitored for survival. All wax moth larvae challenged with the WT or the *bycA*Δ mutant succumbed to mortality within a week. The *cnbR*Δ mutant was avirulent, whereas about 65% of wax moth larvae inoculated with the *bycA*Δ *cnbR*Δ mutant or CnSp4 mutant survived. Results of a log rank (Mantel-Cox) test were statistically significant (*P* < 0.0001). A pairwise comparison was also performed with the following groups: WT versus *bycA*Δ mutant (*P* = 0.82) and WT versus *cnbR*Δ mutant or *bycA*Δ *cnbR*Δ mutant or CnSp4 mutant (*, *P* < 0.0001).

The yeast-locked *cnbR*Δ mutant is less virulent than the wild type in a G. mellonella (wax moth) host (27). To test if calcineurin is involved in virulence, we injected wax moths (*n* = 15/group) with 10,000 spores of the wild type, the *bycA*Δ single mutant, the *cnbR*Δ single mutant, or the *bycA*Δ *cnbR*Δ double mutant or with phosphate-buffered saline (PBS) (mock) and monitored survival for 7 days. As shown in Fig. 7, all of the wax moth larvae challenged with wild-type or *bycA*Δ spores succumbed to infection within a week, while the *cnbR*Δ and *bycA*Δ *cnbR*Δ mutants exhibited a significantly higher survival rate. Interestingly, only about 65% of the wax moth infected with the CnSp4 mutant (spontaneous double mutant) or with the *bycA*Δ *cnbR*Δ mutant survived, while 100% of the wax moth infected with the *cnbR*Δ mutant survived. We also injected wax moth larvae with 10,000 (1×), 20,000 (2×), or 30,000 spores (3×) of the *bycA*Δ *cnbR*Δ double mutant; as shown in Fig. S7, it was only the 3× spore inoculum that achieved 100% mortality.

10.1128/mBio.02949-19.7 FIG S7 A *bycA*Δ *cnbR*Δ double mutant is lethal in a wax moth larva host only at higher inocula. The wax moth larvae (*n* = 15/group) were inoculated with 1 × 10^4^ (1×) to 3 × 10^4^ (3×) *bycA*Δ *cnbR*Δ double mutant or WT spores (1×) in a mixture containing 2 μl PBS via the last left proleg and were monitored for survival. All wax moth challenged with the WT or the *bycA*Δ *cnbR*Δ mutant (3×) succumbed to mortality within a week. A total of 65% of the wax moths inoculated with the *bycA*Δ *cnbR*Δ mutant (1×) and 45% of the wax moths inoculated with the *bycA*Δ *cnbR*Δ mutant (2×) survived. Results obtained with a log rank (Mantel-Cox) test were statistically significant (*P* < 0.0001). A pairwise comparison was also performed with the following groups: WT versus *bycA*Δ *cnbR*Δ mutant (1×) (*, *P* < 0.0001); WT versus *bycA*Δ *cnbR*Δ mutant (2×) (*, *P* = 0.0014); WT versus *bycA*Δ *cnbR*Δ (3×) (*P* = 0.9460). Download FIG S7, TIF file, 0.1 MB. Copyright © 2020 Vellanki et al. 2020 Vellanki et al. This content is distributed under the terms of the Creative Commons Attribution 4.0 International license.

To determine virulence in an immunocompromised murine host, inoculations were performed with the wild-type strain (R7B), the *bycA*Δ single mutant, the *cnbR*Δ single mutant, the *bycA*Δ *cnbR*Δ double mutant, or PBS (mock) via the intratracheal or intravenous route. However, there was no significant difference in survival between the infected groups (Fig. S8). It is interesting that calcineurin mutants were significantly less virulent than the WT in the wax moth larval host but were as virulent as the WT in the murine model.

10.1128/mBio.02949-19.8 FIG S8 *Mucor* virulence in the murine model of systemic and pulmonary mucormycosis. (A) Cyclophosphamide- and cortisone acetate-treated CF1 mice were challenged with 1.5 × 10^6^ spores or yeast cells in a mixture containing PBS via lateral tail vein (systemic mucormycosis model). The animals (*n* = 5/group) were monitored for survival. Results from a log rank (Mantel-Cox) test were not statistically significant (*P* = 0.0585), suggesting no major difference in survival between the WT and calcineurin mutant groups. (B) For the pulmonary mucormycosis model (*n* = 5/group), the same inocula were introduced via intratracheal route and the animals were monitored for survival. Results of a log rank (Mantel-Cox) test was not statistically significant (*P* = 0.1188). Download FIG S8, TIF file, 0.2 MB. Copyright © 2020 Vellanki et al. 2020 Vellanki et al. This content is distributed under the terms of the Creative Commons Attribution 4.0 International license.

## DISCUSSION

Calcineurin is conserved widely across pathogenic fungi and is involved in cell wall integrity, morphogenesis, and virulence (20, 21, 61). We have previously shown that in the presence of a calcineurin inhibitor (FK506), *Mucor* exhibits yeast morphology even under aerobic conditions; however, in strains that are resistant to FK506, *Mucor* grows as hyphae (27). FKBP12 is the drug receptor for FK506, and when bound, the FKBP12-FK506 complex inhibits the phosphatase activity of calcineurin by binding to the interface of the calcineurin regulatory and catalytic subunits (45). One of the common ways through which most pathogenic microbes become resistant to drugs is that of having mutations in the drug receptor, in this case, FKBP12 or the catalytic A subunit or regulatory B subunit of calcineurin (27). Calo et al. have shown that *Mucor* can exhibit transient resistance to FK506 by triggering RNA interference specific to the gene encoding FKBP12, resulting in silencing of the drug target gene (51, 52). In this study, we identified a novel resistance mechanism through which *Mucor* can also become resistant to calcineurin inhibitors. Whole-genome sequencing (WGS) and targeted sequencing of the genomes of spontaneous mutants (CnSp and CSR mutants) revealed DNA sequence alterations in the *bycA* locus in 31 of 36 resistant isolates (Table 1 and Table 2).

### A novel link between calcineurin and BycA, an amino acid permease in *Mucor*.

On the basis of our sequencing analysis, we hypothesized that deletion of *bycA* should result in stable resistance to calcineurin inhibitors. Indeed, *bycA*Δ mutants are resistant to FK506. Unlike *cnbR*Δ mutants, which grow as yeast, cells of the *bycA*Δ *cnbR*Δ mutant fully grow as hyphae such as were seen with the WT (Fig. 3). This verifies that *Mucor* can become resistant to calcineurin inhibitors as a consequence of the presence of a second mutation in the *bycA* gene. *Mucor* has a secondary conserved mechanism that promotes hyphal growth even in the absence of a functional calcineurin. Interestingly, however, we did not observe Mendelian DNA mutations in the *bycA* locus in 5 (of 36) of the mutants on which we performed sequencing even though they exhibited the same phenotype as the remaining suppressors; one possibility is that *Mucor* may also produce small RNAi targeting *bycA* or that there could be another mechanism(s) that confers *Mucor* resistance against calcineurin inhibitors. Future studies are required to elucidate these mechanisms further.

### How is BycA linked to calcineurin and morphogenesis?

It is not known if BycA is a direct posttranslation modification target of calcineurin. However, our data suggest that calcineurin negatively regulates the expression of BycA at the mRNA level. It is also possible that calcineurin is involved in the stability of the *bycA* mRNA. Further investigation is ongoing to elucidate how calcineurin regulates expression of BycA. Interestingly, Chow et al. revealed that, in Cryptococcus neoformans, a gene encoding a putative amino acid permease (CNAG_01118) is overexpressed when calcineurin is deleted and that this is independent of the presence of Crz1, a well-known target of calcineurin (62). Whether deletion of the amino acid gene would result in suppression of calcineurin mutant phenotypes remains to be tested in C. neoformans.

### BycA as a missing link between calcineurin and PKA.

Studies by us and others have shown that *Mucor* morphology is primarily dependent on (i) respiratory conditions and (ii) calcineurin and PKA activity (27–30, 37–39, 41). *Mucor* exhibits hyphal growth under aerobic conditions. Under anaerobic conditions with high CO_2_, *Mucor* grows as yeast and has PKA activity that is increased via the bicarbonate-cAMP pathway (27, 28, 30). We have previously shown that even under aerobic conditions when calcineurin function is inhibited by FK506 or absent in the *cnbR*Δ mutant, *Mucor* grows as a yeast and PKA activity remained high (27). The bicarbonate-cAMP pathway is likely not involved under aerobic conditions as the carbon dioxide levels are very low. It has been suggested that there is an antagonistic relationship between calcineurin and PKA in fungal systems in Ustilago maydis and S. cerevisiae (63, 64).

In this study, we found that calcineurin and PKA are inversely related through BycA and that the overall PKA activity was lower in wild-type hyphae with lower *bycA* expression but higher in yeast with higher *bycA* expression (Fig. 4A and C). PKA activity also remained lower in the *bycA*Δ *cnbR*Δ double mutant because even though calcineurin function was suppressed, the double mutant did not have BycA function to activate PKA and promote yeast growth. For the same reason, the *bycA*Δ *cnbR*Δ double mutants exhibited hyphal morphology. We also found that the amino acid permease BycA was able to activate PKA without a requirement for cAMP. Our finding that BycA can activate PKA is congruent with previous findings in C. albicans and S. cerevisiae, where the general amino acid permease Gap1 can activate PKA in a cAMP-independent manner (57, 58). It is possible that this is achieved through an imbalance between the catalytic and regulatory subunits of PKA. It was previously reported that kelch repeat homolog proteins 1 and 2 (Krh1 and Krh2) promote the association between PKA catalytic and regulatory subunits in S. cerevisiae and that deletion of *krh1*/*2* leads to a lower cAMP requirement for PKA activation (65). Since Krh proteins are evolutionarily conserved in eukaryotes, it is possible that such a mechanism may also exist in *Mucor.* Our future studies will further focus on determining how BycA activates PKA in *Mucor*.

Our previous studies showed that cellular PKA activity levels were elevated when calcineurin was inhibited in C. neoformans and Rhizopus delemar (27). How this link is achieved in these fungi remains to be elucidated. There is evidence that higher expression of an amino acid permease in C. neoformans is correlated with the phenotypes regulated by PKA; treatment with urea resulted in a 27-fold increase in the level of an amino acid permease (CNAG_01118) and in increased capsule production, known to be regulated by PKA (66–68). Our current study investigated the links between calcineurin, amino acid permease, and PKA which could also exist in C. neoformans and possibly other fungi.

### Calcineurin governs virulence and plays important roles in host-pathogen interactions in *Mucor*.

Studies of several pathogenic fungi have shown that morphology is linked with expression of virulence factors, colonization of the host, evasion of host immune responses, etc. (69, 70). The *cnbR*Δ mutants were shown to be significantly less virulent in a heterologous wax moth larva host model (27); however, it was not clear if the diminished virulence potential of *cnbR*Δ mutants was due to morphology or loss of the calcineurin or both. In the current study, using a hyphal growth strain lacking calcineurin (*bycA*Δ *cnbR*Δ double mutant), we identified a novel downstream function of calcineurin that is independent of its function in governing morphology and that contributes to *Mucor*-host interactions and virulence. For example, macrophages challenged with mutants that lack calcineurin displayed significantly higher phagosome maturation than macrophages challenged with the wild type, irrespective of their morphology (Fig. 5). The ability of *Mucor* to cause damage to the endothelium was dependent upon the presence of calcineurin (Fig. 6A).

Angiogenesis is the process by which new blood vessels arise from preformed blood vessels. Proteins such as fibroblast growth factor-2 (FGF-2) and vascular endothelial growth factor (VEGF) promote angiogenesis (71). We have previously shown that the ability of C. albicans to induce FGF-2 is morphology dependent, as nonfilamentous strains of C. albicans failed to induce FGF-2 (72). We have also shown that only the WT and not *cnbR*Δ mutants induced a host FGF-2 response in *Mucor* (43). In this study, we further found that a *bycA*Δ *cnbR*Δ double mutant also failed to induce FGF-2 (Fig. 6B). Our data suggest that the ability of *Mucor* to induce host FGF-2 is dependent on a novel downstream function of calcineurin which is independent of its function in regulating morphology. Studies by us and others have shown that candidalysin from C. albicans and gliotoxins from Aspergillus fumigatus regulate the host FGF-2 response (72, 73). Hence, it is possible that toxins from *Mucor* (74, 75) also regulate host FGF-2 response. We are currently working on identifying the *Mucor* factor(s) that facilitates *Mucor* interactions with endothelial cells to induce FGF-2 response.

Larvae of Galleria mellonella have largely been used to study virulence and for testing antifungal drugs (27, 47, 74). Wax moth larvae possess an innate immune system that is both structurally and functionally similar to that of mammals at the humoral and cellular levels (76). We have previously shown that *cnbR*Δ mutants are avirulent in wax moth larva host (27). In this study, we found that *bycA*Δ *cnbR*Δ double mutants are also less virulent (Fig. 7). It is congruent that more wild-type spores survived than *cnbR*Δ yeast during interactions with bone marrow murine macrophages (see Fig. S9 in the supplemental material).

10.1128/mBio.02949-19.9 FIG S9 Survival of wild-type spores and *cnbR*Δ yeast during cocultures with bone marrow macrophages. The spores survived the macrophages at significantly higher levels than were seen with the yeast-locked mutant. The experiment was performed as described previously (K.-W. Jung, Y. Lee, E. Y. Huh, S. C. Lee, et al., mBio 10:e01726-18, 2019, https://doi.org/10.1128/mBio.01726-18). Download FIG S9, TIF file, 0.1 MB. Copyright © 2020 Vellanki et al. 2020 Vellanki et al. This content is distributed under the terms of the Creative Commons Attribution 4.0 International license.

Interestingly, unlike in the wax moth host system, the *cnbR*Δ mutant also caused significant mortality like that seen with the wild type in the pulmonary and systemic murine model of mucormycosis. This observation may be due to the neutropenic host conditions caused by treatment with cyclophosphamide and cortisone. Calcineurin is required to escape innate immune cells by blocking phagosome maturation; however, lack or significant lower numbers of phagocytic cells resulted in higher mortality by the yeast-locked *cnbR*Δ mutant. In addition, treatment with cyclophosphamide and cortisone acetate could impact the phagocytic potential of the innate immune cells, as it was previously shown that treatment with cortisone acetate resulted in failure of alveolar macrophages to inhibit germination of Rhizopus oryzae (77). Alternatively, *Mucor* yeast is more immunogenic than *Mucor* spores and therefore the yeast might have caused mortality in a different manner than the spores (43). This could represent an analogy to the *Cryptococcus rim101* mutants (78). The *rim101* mutant lacks virulence traits and yet is more immunogenic than the wild type; nevertheless, the mutants still caused mortality in a murine infection model via a substantial host immune response. Further study is required to determine if *Mucor cnbR*Δ yeast would cause hyperimmune responses in a murine lung infection model.

**FIG 8:**
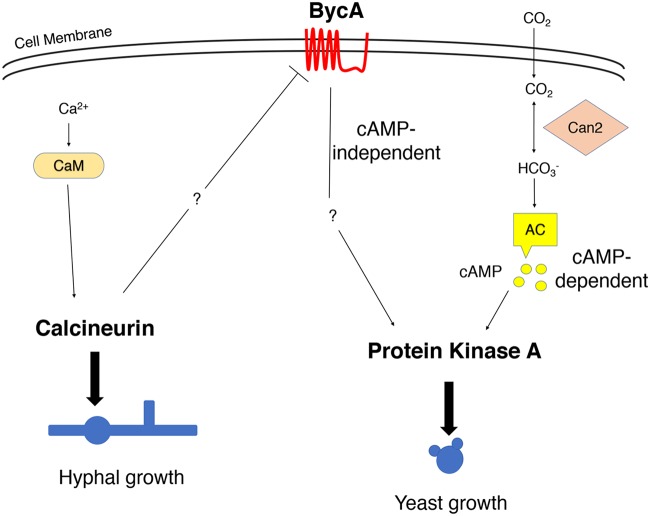
Calcineurin, BycA, and PKA in the morphogenesis of *Mucor* under aerobic conditions. Calcineurin is the master regulator of *Mucor* morphology. Active calcineurin positively regulates hyphal growth and negatively regulates yeast growth. This is achieved by suppressing the expression of the *bycA* gene, thereby preventing an increase in PKA activity. Calcium-calmodulin activates calcineurin to promote hyphal growth under aerobic conditions. However, when calcineurin is not functional, *bycA* gene expression is significantly elevated. BycA then activates PKA in a cAMP-independent manner to promote yeast growth. Under anaerobic conditions with high CO_2_ levels, PKA is activated through the CO_2_-cAMP pathway. Neither BycA nor calcineurin has a defined role in the regulation of *Mucor* morphology under anaerobic conditions. Can2, carbonyl anhydrase; AC, adenylyl cyclase; CaM, calmodulin.

In summary, we identified a novel mechanism through which *Mucor* can become resistant to calcineurin inhibitors, where loss-of-function mutations in the *bycA* gene confer resistance against calcineurin inhibitors. This resistance mechanism is achieved by BycA regulating the activity of protein kinase A via a cAMP-independent pathway that has yet to be elucidated (Fig. 8). As calcineurin is a major virulence factor in many pathogenic fungi, it is worth investigating if this relationship between calcineurin, BycA, and PKA is also conserved in other pathogenic fungal systems. Calcineurin also governs key *Mucor*-host interactions and is an attractive target for developing antifungals to treat mucormycosis.

## MATERIALS AND METHODS

### Ethics statement.

All animal experiments were conducted at the University of Texas at San Antonio (UTSA) in accordance with the Institutional Animal Care and Use Committee (IACUC) guidelines and in full compliance with the United States Animal Welfare Act (Public Law 98-198) and National Institutes of Health (NIH) guidelines. The animal protocol (MU104) used in this study was approved by the UTSA IACUC. The experiments were conducted in the Division of Laboratory Animal Resources (DLAR) facilities, which are accredited by the Association for Assessment and Accreditation of Laboratory Animal Care (AAALAC).

### Fungal strains and growth conditions.

All fungal strains and plasmids used in this study are listed in Table S1A in the supplemental material. For spore production, *Mucor* strains were inoculated and maintained on yeast extract-peptone-glucose (YPG; 3 g/liter yeast extract, 10 g/liter peptone, 20 g/liter glucose, 2% agar, pH 4.5) or yeast extract-peptone dextrose (YPD; 10 g/liter yeast extract, 20 g/liter peptone, 20 g/liter glucose, 2% agar, pH 6.5) agar at 26°C or 30°C for 4 days. *cnbR*Δ mutants were maintained on YPD agar at 30°C. For *in vitro Mucor-*host interaction studies or *in vivo* survival studies, *cnbR*Δ mutants were grown overnight in liquid YPD at 30°C with shaking. For high-CO_2_ conditions, the flasks were entirely filled with YPD broth, and the wild type (R7B) was inoculated on the bottom of the flasks. The flasks were sealed with Parafilm and were left at room temperature overnight without being disturbed. For testing with calcineurin inhibitors, *Mucor* strains were grown in liquid YPD or on YPD agar plates supplemented with FK506 (Astellas Pharma Inc.) (1 μg/ml) or CsA (LC Laboratories) (2 or 100 μg/ml) at 30°C for 2 to 5 days.

10.1128/mBio.02949-19.10 TABLE S1 (A) List of strains used in the study. (B) List of primers used in the study. Download Table S1, DOCX file, 0.03 MB. Copyright © 2020 Vellanki et al. 2020 Vellanki et al. This content is distributed under the terms of the Creative Commons Attribution 4.0 International license.

### Generation of suppressor mutants CnSp and CSR.

For generation of CnSp mutants, 10^3^ cells of *cnbR*Δ mutants were spotted on YPD agar and grown at 30°C for 5 or more days. Hyphal sectors emerging from yeast colonies were propagated on fresh YPD plates. For CSR mutant generation, *cnaB*Δ mutants were grown on YPD agar containing CsA (2 μg/ml) for 5 days or longer, and the hyphal colonies were propagated further.

### Whole-genome sequencing and targeted sequencing of CnsP and CSR mutants.

Genomic DNA of the 12 spontaneous mutant strains and the MU402 strain was used for sequencing. A paired-ended Truseq library was constructed with the genomic DNA and sequenced on an Illumina HiSeq 2000 platform at the University of North Carolina at Chapel Hill School of Medicine. The reads were mapped to the genome of CBS277.49 (79) using the short-read component of the Burrows-Wheeler Aligner (BWA) (80). SNP calling was performed using the Genome Analysis Toolkit (GATK ver. 2.4-9) pipeline and the Unified Genotyper with the haploid setting (81). Whole-genome seqeunce data were deposited at the NCBI Sequence Read Archive (SRA) under accession number PRJNA597331.

### Disruption of genes.

The primers used in the study are listed in Table S1B. To disrupt the *bycA* gene, nearly 1-kb upstream and downstream sequences flanking the *bycA* gene were PCR amplified (Phusion High-Fidelity DNA polymerase; NEB) using primers SL3 and SL4 and primers SL7 and SL8, respectively. The *pyrG*-*dpl237* marker (53) was amplified using SL5 and SL6 primers. All three fragments were gel excised, and 75 ng of each fragment was amplified using an overlap PCR strategy with primers SL9 and SL10 such that the marker was placed between the flanking regions. The product was cloned into a TOPO vector (pSL26), represent minus signs, please specify what the which was then linearized using SmaI enzyme and transformed into *Mucor* strain Mu402 (*pyrG leuA* double mutant) via electroporation (82). The transformants were selected on minimal media at pH = 3.2 with Casamino Acids (MMC; 10 g Casamino Acids, 0.5 g yeast nitrogen base without amino acids and ammonium sulfate, 20 g glucose, 1 mg niacin, 1 mg thiamine, 15 g agar, 1 liter of distilled water [dH_2_O]) containing 0.5 M sorbitol as described previously (82). Two independent transformants (MSL47.1 and MSL47.2; Table S1A) of 12 were positive for disruption of *bycA.*

For disruption of the *cnbR* gene, 5′ and 3′ flanking regions were amplified using SL243 and SL281 and using SL282 and SL244, respectively. A *leuA* marker was amplified using SCL737 and SCL738. Overlap PCR was performed using primers SCL286 and SCL287. The pSL58 plasmid containing the disruption cassette was linearized and used to transform MSL47.2. The transformants were selected on YNB medium (1.5 g ammonium sulfate, 1.5 g glutamic acid, 0.5 g yeast nitrogen base without amino acids and ammonium sulfate, 10 g glucose, 20 g agar, 1 liter dH_2_O). Two of 18 independent transformants (MSL68.1 and MSL68.2) were positive for disruption of *cnbR*.

### Reverse transcriptase quantitative PCR (RT-qPCR).

Based on the ORF sequences, we designed primers for *bycA* (SL346 and SL347) and *cnbR* (SCL 578 and SCL 579) that span across two exons. *Mucor* strains were grown overnight in YPD liquid medium, and total RNA was isolated the next day using TRIzol reagent according to the manufacturer’s instructions. 1 μg of RNA from each sample was used for cDNA synthesis (High Capacity cDNA reverse transcription kit with RNase inhibitor; Applied Biosystems), and qPCR was performed using SYBR green QPCR master mix (Thermo Scientific). The actin gene (primers SCL368 and SCL369) served as an internal control. Three independent replications were performed for each experiment with RNA obtained from two independent preparations.

### Protein kinase A activity assay.

*Mucor* strains were grown under appropriate conditions overnight, and collection of crude protein extracts and overall PKA activity measurement were performed using a DetectX PKA (protein kinase A) activity kit according to the manufacturer’s instructions. Briefly, crude protein extracts were obtained by subjecting the samples to activated cell lysis buffer with protease inhibitor cocktail, phenylmethylsulfonyl fluoride (PMSF), and sodium orthovanadate. Each sample was vigorously shaken in a bead beater (five times for 1 min each time with 1 min of cooling) followed by centrifugation to collect supernatants containing crude extracts. Total protein was quantified using the Bradford method (Bio-Rad). A 0.5-μg volume of each sample was used to determine overall PKA activity according to the manufacturer’s instructions.

### cAMP determination.

*Mucor* strains were grown under appropriate conditions overnight, and cells corresponding to 60 mg (wet weight) were flash frozen and immersed in 0.1 M HCl the next day. Crude extracts were obtained as described above. cAMP levels were measured according to the manufacturer’s instructions (Direct cAMP enzyme-linked immunosorbent assay [ELISA] kit; Enzo).

### Phagosome maturation assay.

Murine macrophage cell line J774.A1 was maintained in Dulbecco’s modified Eagle medium (DMEM) with 10% fetal bovine serum (FBS) and a 200 U/ml mixture of penicillin and streptomycin antibiotics at 37°C and 5% CO_2_. Upon confluence, a cell scraper was used to detach cells, and 5 × 10^5^ cells were plated in each well of a 24-well glass plate. After 24 h, *Mucor* spores or yeast cells were added at a multiplicity of infection (MOI) of 1 along with 10 μM LysoTracker Green DND-26 and 1 μg/ml Hoechst 33342 stain. A Zeiss Axio Observer microscope was used 30 min later to image fields containing macrophages and *Mucor* cells. The experiment was performed in triplicate and was repeated on two different occasions.

### LDH assay and FGF-2 ELISA.

Primary human umbilical vein endothelial cells (HUVECs) were purchased from Lonza and were maintained in endothelial basal medium (EBM) containing hydrocortisone, ascorbic acid, insulin growth factor, heparin, and FBS at 37°C and 5% CO_2_ according to the manufacturer’s instructions. Confluent cells were trypsinized, and 5 × 10^3^ cells/well were seeded in a 96-well plate. After 24 h, 5 × 10^4^ of the appropriate fungal cells in a mixture containing PBS (MOI =10) or an equal volume of PBS was added to each well. At 24 h postinfection, the supernatant was collected to quantify LDH levels (CytoTox 96 nonradioactive cytotoxicity assay; Promega) or FGF-2 protein levels using ELISA (R&D Systems). The level of LDH release was calculated as described previously (83).

### Virulence test.

For production of *Mucor* spores, appropriate strains were grown on YPG agar at 26°C for 4 days under light. The *cnbR*Δ mutant was grown overnight in YPD broth at 30°C with aeration. Both the spores and yeast were washed two times with PBS, and different inoculums ranging from 1 × 10^4^ to 3 × 10^4^ cells in a mixture with 2 μl PBS were injected into the wax moth host through the last left proleg. Differences between the survival curves were evaluated for significance using the Kaplan-Meier test. The experiment was performed on two different occasions with *n* = 15 animals for each group.

Six-week-old CF1 mice were immunocompromised with cyclophosphamide (250 mg/kg of body weight via the intraperitoneal route) and cortisone acetate (500 mg/kg via the subcutaneous route) every 5 days starting 2 days before inoculation (84). On the day of inoculation, the mice were anaesthetized using isoflurane and 1.5 × 10^6^ spores or yeast cells in a mixture containing PBS were introduced via the intratracheal route (pulmonary), followed by routine monitoring. Two independent experiments were performed with *n* = 5 for each group. Data represent results from a single experiment.

### Statistics.

Prism (Version 7; GraphPad Software Inc.) was used to perform statistical analysis. A *P* value of ≤0.05 was considered significant.

### Data availability.

Whole-genome seqeunce data were deposited at the NCBI Sequence Read Archive (SRA) under accession number PRJNA597331.
