# Supplementary figures and images for: A Novel Resistance Pathway for Calcineurin Inhibitors in the Human-Pathogenic Mucorales Mucor circinelloides
Source: mBio. 2020 Jan 28;11(1):e02949-19. doi: 10.1128/mBio.02949-19 (PMC6989107; doi:10.1128/mBio.02949-19)

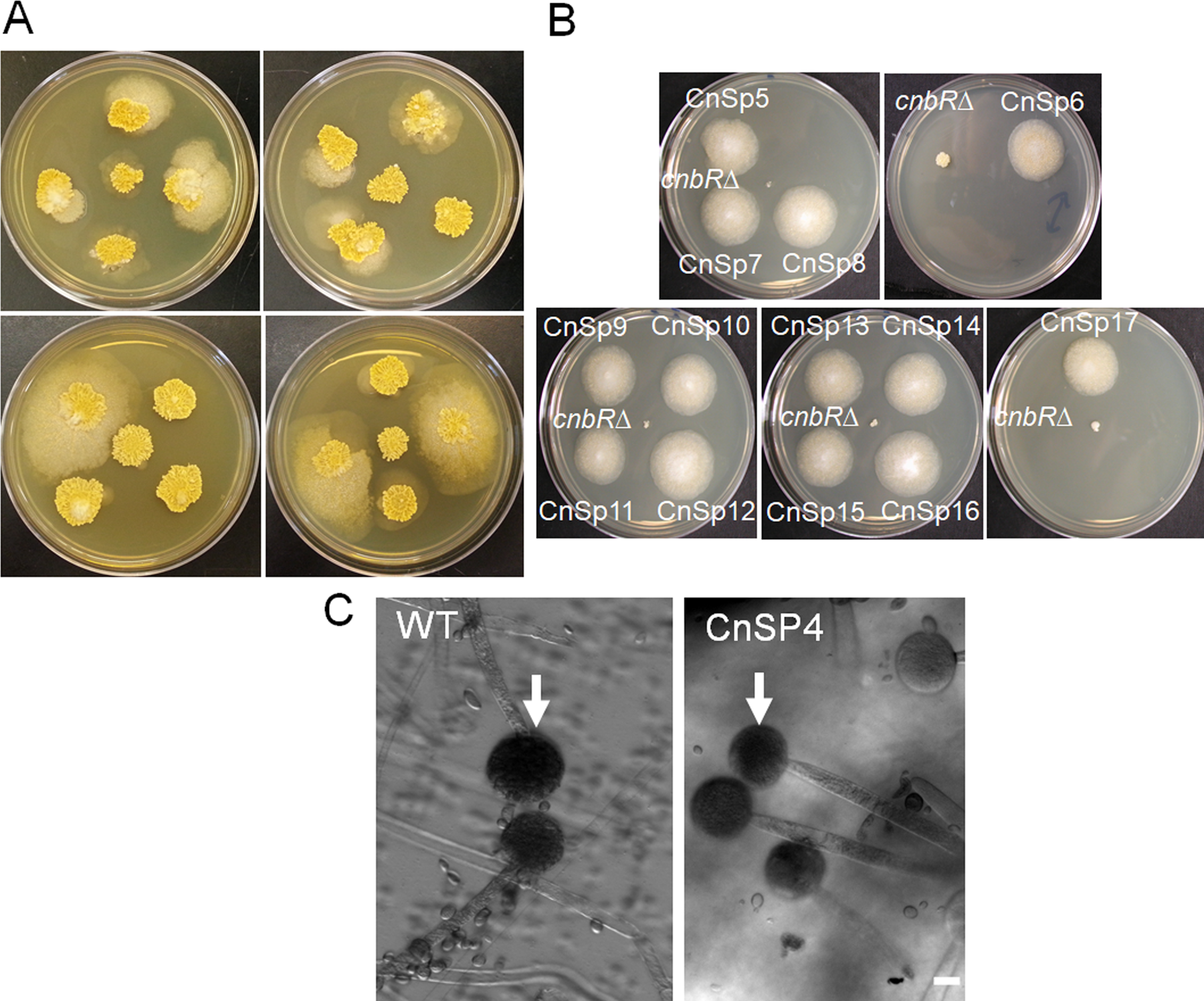

Supplement: FIG S1 [file mBio.02949-19-sf001.tif]

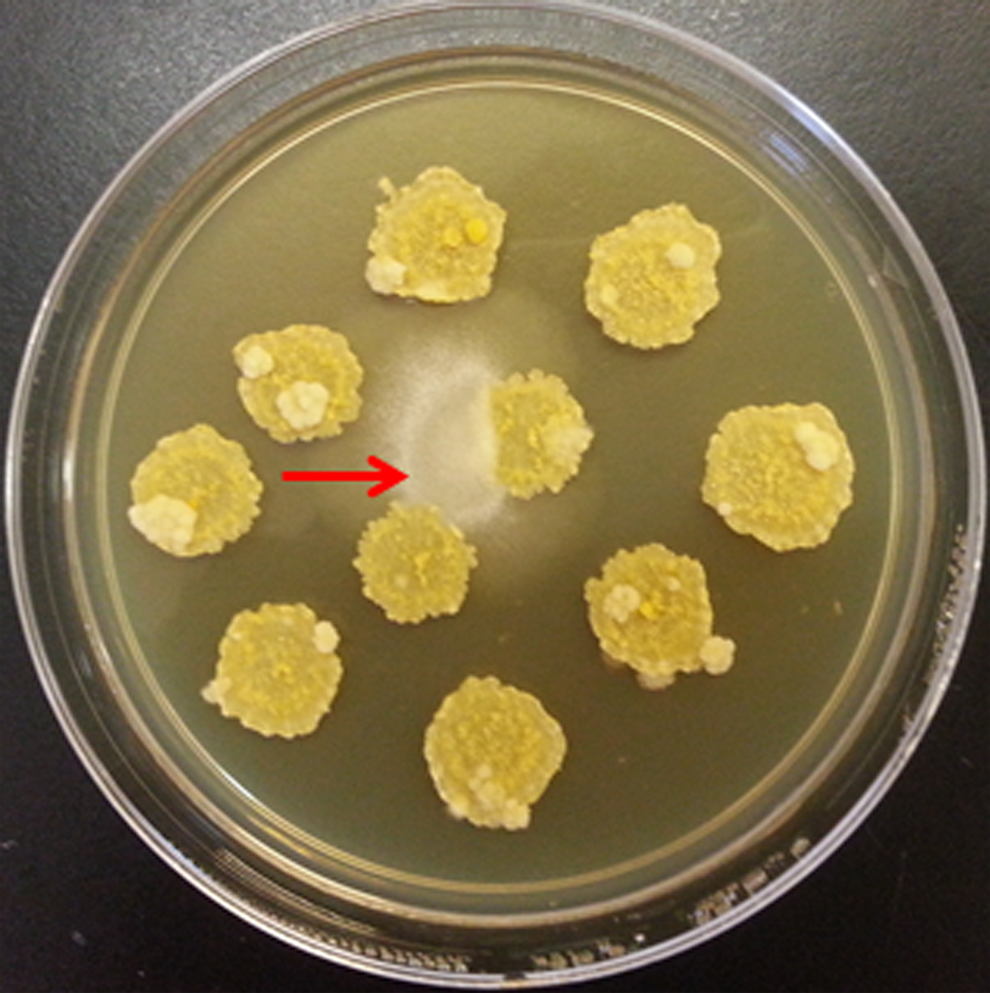

Supplement: FIG S2 [file mBio.02949-19-sf002.tif]

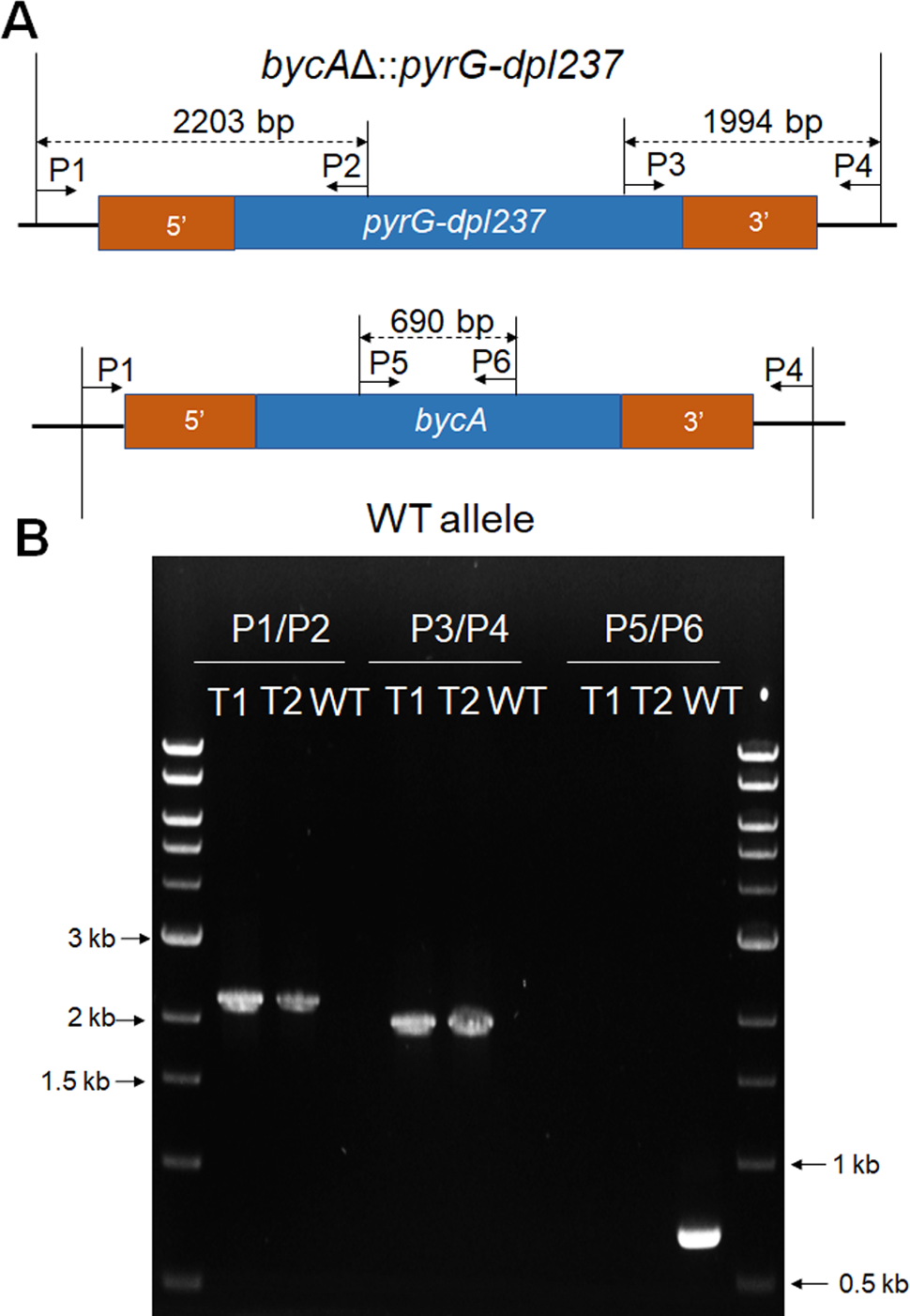

Supplement: FIG S3 [file mBio.02949-19-sf003.tif]

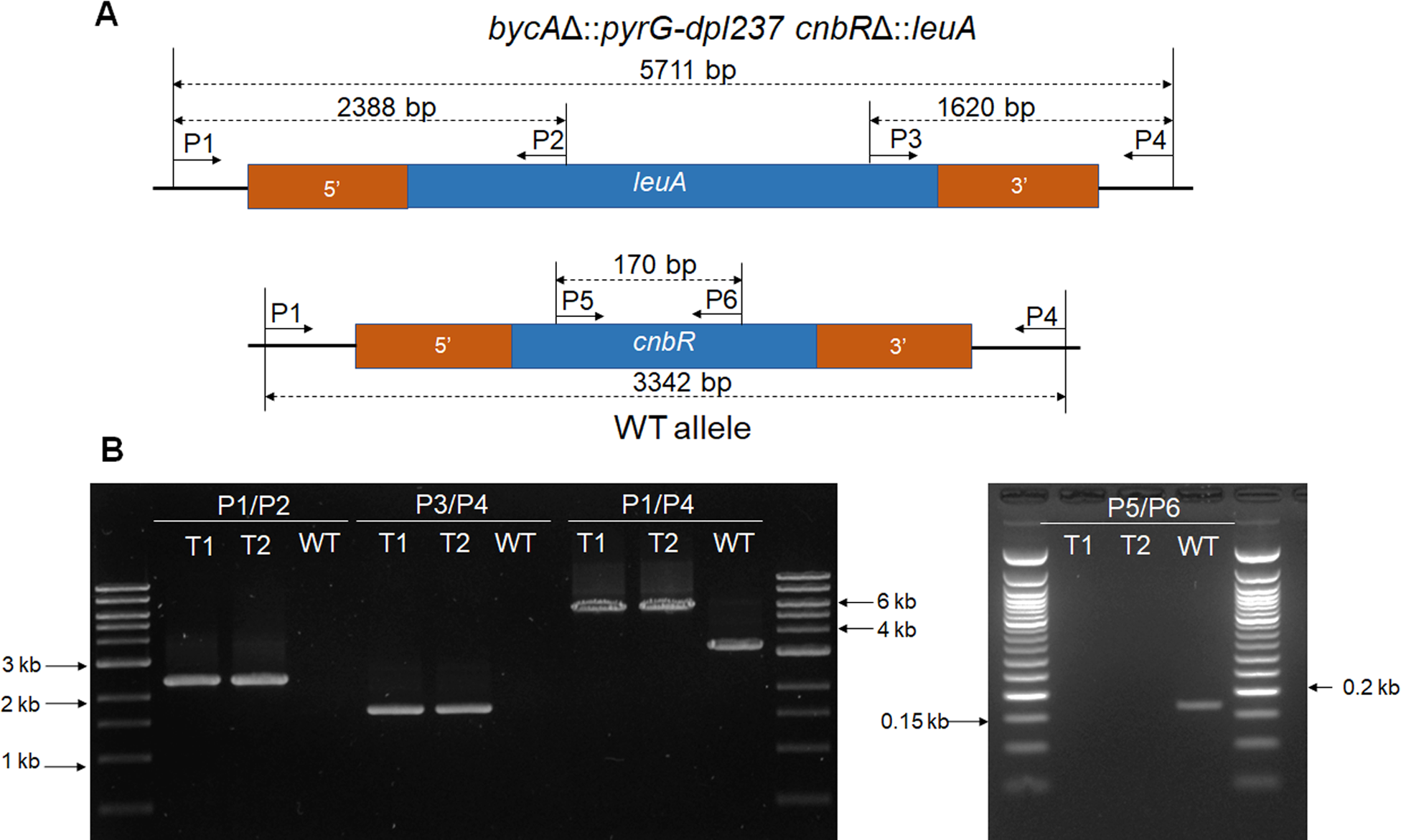

Supplement: FIG S4 [file mBio.02949-19-sf004.tif]

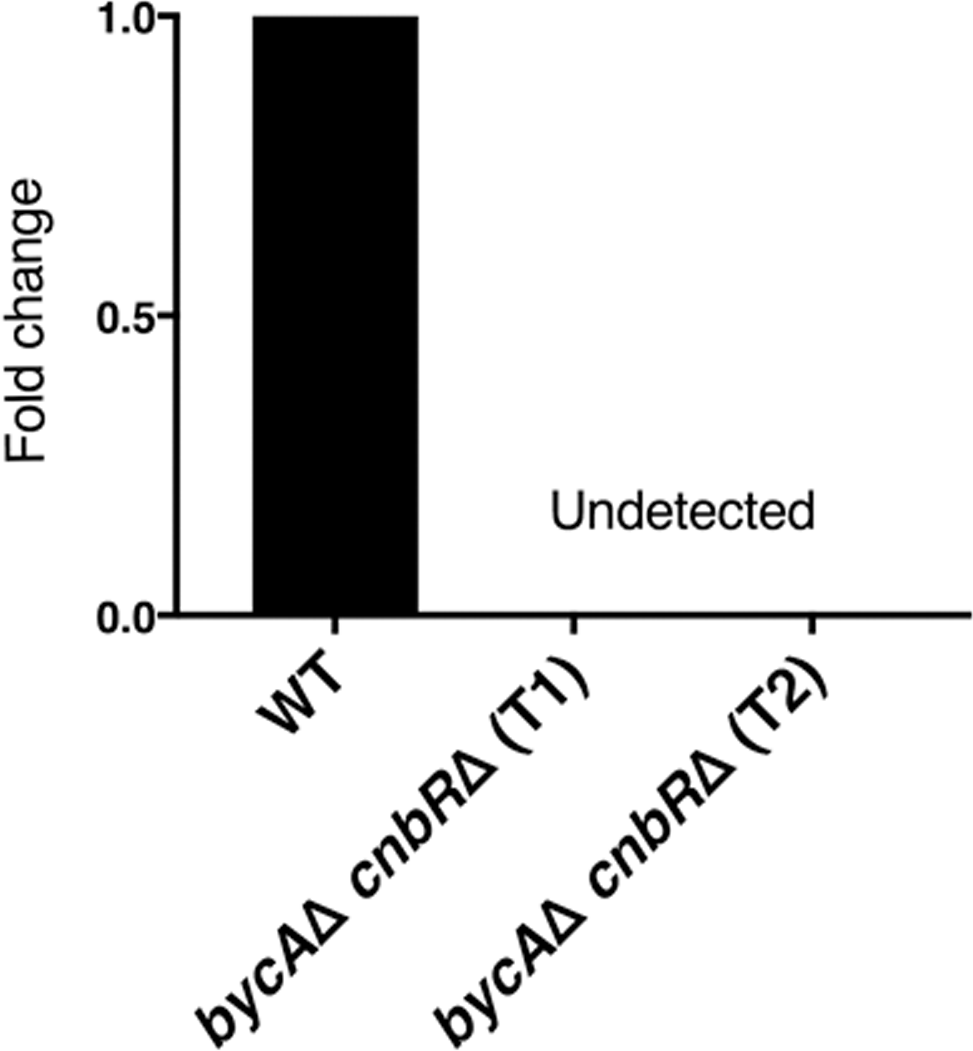

Supplement: FIG S5 [file mBio.02949-19-sf005.tif]

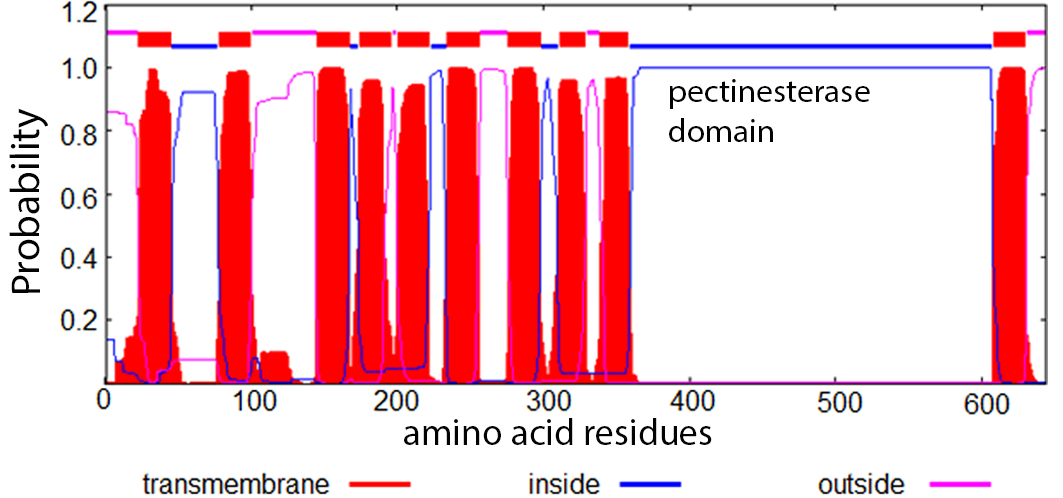

Supplement: FIG S6 [file mBio.02949-19-sf006.tif]

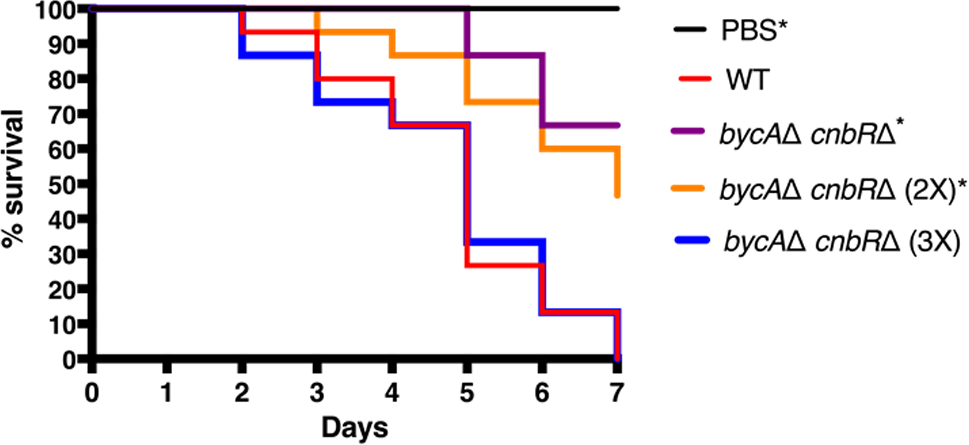

Supplement: FIG S7 [file mBio.02949-19-sf007.tif]

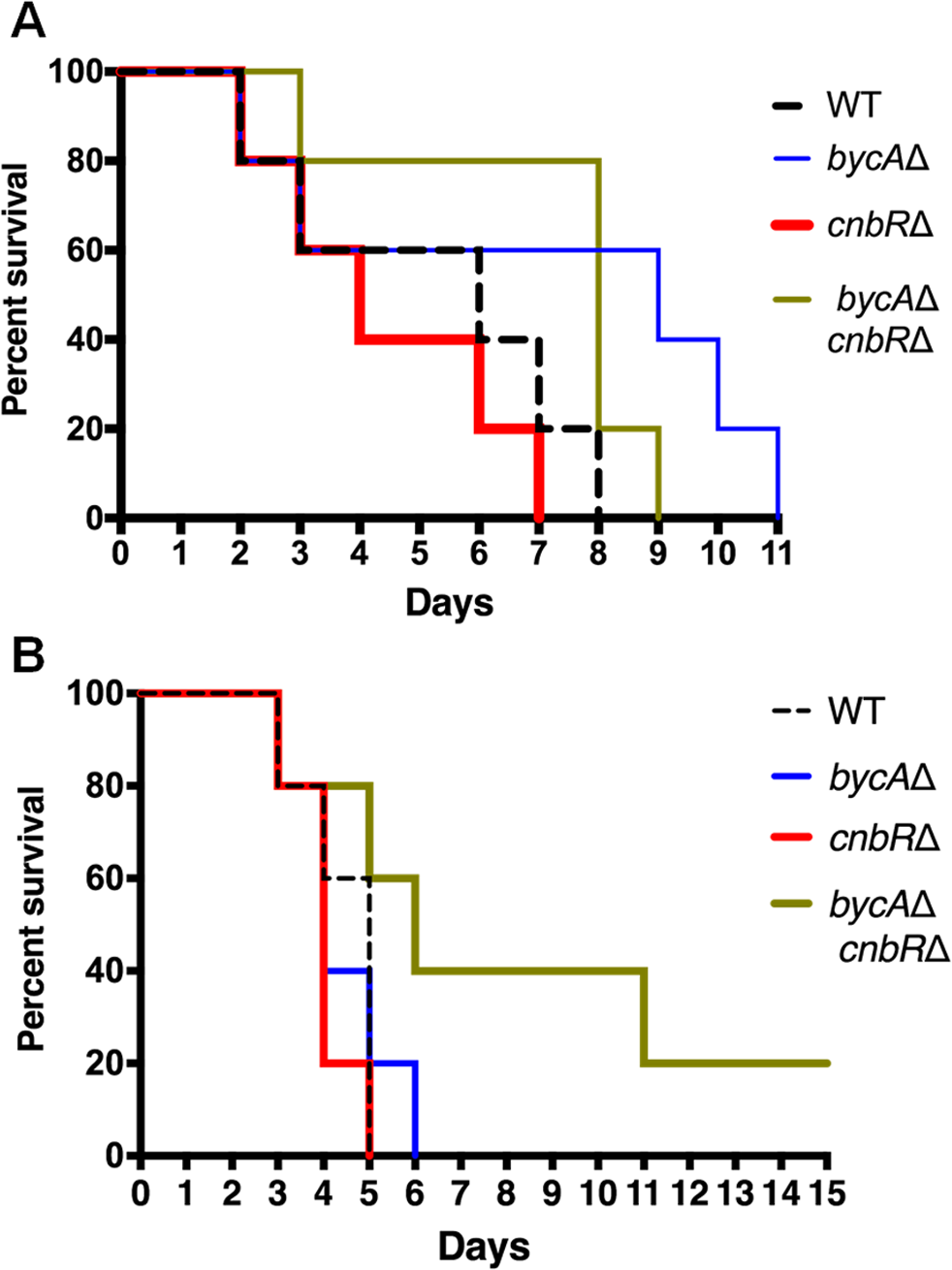

Supplement: FIG S8 [file mBio.02949-19-sf008.tif]

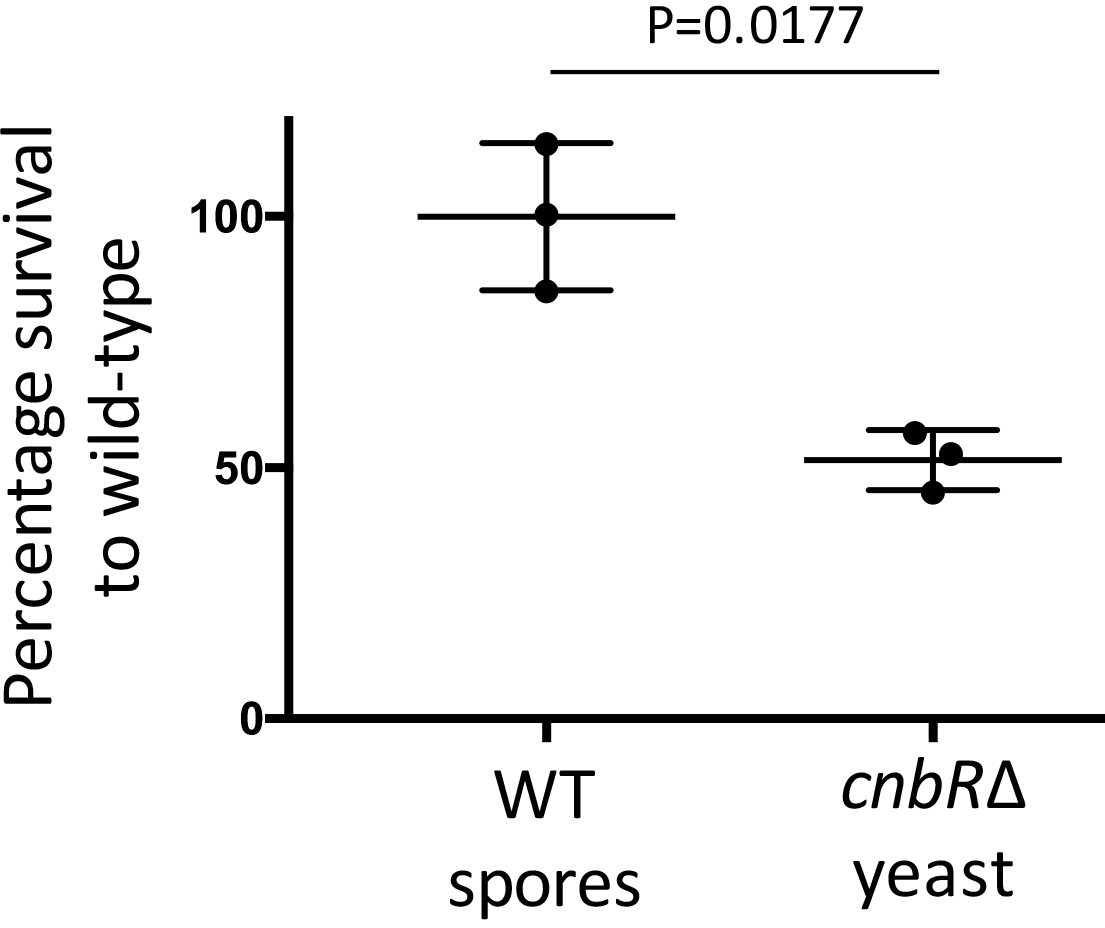

Supplement: FIG S9 [file mBio.02949-19-sf009.tif]
