# Supplementary material for: A Novel Resistance Pathway for Calcineurin Inhibitors in the Human-Pathogenic Mucorales Mucor circinelloides
Source: mBio. 2020 Jan 28;11(1):e02949-19. doi: 10.1128/mBio.02949-19 (PMC6989107; doi:10.1128/mBio.02949-19)
Supplement: TABLE S1 [file mBio.02949-19-st001.docx]

**Table S1.** A) Strains used in this study.

|  | **Strain name** | **Genotype** | **Reference** |
| --- | --- | --- | --- |
| *Mucor circinelloides* | CBS277.49 | Wild-type, *M. circinelloides f. lusitanicus* |  |
|  | R7B | CBS 277.49 background, *leuA^-^* | Joint Genome Institute |
|  | MU402 | CBS 277.49 background, *pyrG^-^ leuA^-^* | (1) |
|  | MSL7 | MU402 origin, yeast-locked mutant, *cnbR*Δ::*pyrG pyrG^−^ leuA^−^* | (2) |
|  | MSL8 | MU402 origin, *cnbR*Δ::*pyrG pyrG^−^ leuA^−^* | (2) |
|  | MSL21 | MU402 origin, *cnaB*Δ::*pyrG pyrG^−^ leuA^−^* | (3) |
|  | MSL22 | MU402 origin, *cnaB*Δ::*pyrG pyrG^−^ leuA^−^* | (3) |
|  | MSL47.1 | MU402 origin, *bycA*Δ::*pyrG-dpl237 pyrG^−^ leuA^−^ pyrG^−^ leuA^−^* | This study |
|  | MSL47.2 | MU402 origin, *bycA*Δ::*pyrG-dpl237 pyrG^−^ leuA^−^ pyrG^−^ leuA^−^* | This study |
|  | MSL68.1 | MU402 origin, *bycA*Δ::*pyrG-dpl237 cnbR*Δ::*leuA pyrG^−^ leuA^−^* | This study |
|  | MSL68.2 | MU402 origin, *bycA*Δ::*pyrG-dpl237 cnbR*Δ::*leuA pyrG^−^ leuA^−^* | This study |
|  | CSR mutants | calcineurin bypass | This study; see Table1 |
|  | CnSp mutants | calcineurin bypass | This study; see Table1 |
| Plasmids | pSL13 | *pyrG* blaster marker in pCR21-TOPO | (4) |
|  | pSL19 | *leuA* marker in pCR blunt II TOPO | This study |
|  | pSL26 | *bycA*∆ disruption allele in pCR blunt II TOPO | This study |
|  | pSL58 | *cnbR*∆ disruption allele in pCR blunt II TOPO | This study |

**Table S1.** B) Primers used in this study.

| Name | 5’ to 3’ sequence | Remark |
| --- | --- | --- |
| SL3 | GCCAGCACAGAATAGCAACA | Primer for *bycA* disruption, 5’ region |
| SL4 | CTGGCCGTCGTTTTACGCTTCCAATGCCCTTTACTG | Primer for *bycA* disruption, 5’ region |
| SL5 | GTAAAACGACGGCCAG | Primer for *bycA* disruption, *pyrG – dpl237* |
| SL6 | CAGGAAACAGCTATGAC | Primer for *bycA* disruption, *pyrG – dpl237* |
| SL7 | GTCATAGCTGTTTCCTGTAGACCGCTTCATTGCCTTT | Primer for *bycA* disruption, 3’ region |
| SL8 | GCCCAAGTGCAATACGAGAT | Primer for *bycA* disruption, 3’ region |
| SL9 | GCACCCTCTGCCATTACTGT | Nested forward primer for *bycA* disruption |
| SL10 | TTGCTGCAATGCGCTTATAC | Nested reverse primer for *bycA* disruption |
| SCL566 | ACCCACTCACTTTCCATTCG | Primer for *pyrG* to confirm *bycA* disruption |
| SCL567 | TGCTTTTGTTGGCTGAGATG | Primer for *pyrG* to confirm *bycA* disruption |
| SL182 | CAGCCTTTTAACGGGGTACA | Primer within the *bycA* ORF |
| SL183 | AAACCGCTTTTGTCCATCAC | Primer within the *bycA* ORF |
| SL346 | GACCACCATTGCTCAGGTCT | RT PCR primer for *bycA* |
| SL347 | ACCATCCACCAGGATAGACG | RT PCR primer for *bycA* |
| SCL368 | ATGGTCGGTATGGGTCAAAA | RT PCR primer for actin |
| SCL369 | GCCTCAGTCAAGAGGACAGG | RT PCR primer for actin |
| SL243 | GCCTTGGTCAAGGAGTTGAA | Primer for *cnbR* disruption, 5’ region |
| SL281 | GTAGGGAGCACCGATGATGTAGTTGCGAACCAGATTGACC | Primer for *cnbR* disruption, 5’ region |
| SCL737 | ACATCATCGGTGCTCCCTAC | Primer for *cnbR* disruption, *leuA* |
| SCL738 | AGCGTCATGTTCAGGAAAGAG | Primer for *cnbR* disruption, *leuA* |
| SL282 | GTAGGGAGCACCGATGATGT AGTTGCGAACCAGATTGACC | Primer for *cnbR* disruption, 3’ region |
| SL244 | CATGACCACACCCACACAAT | Primer for *cnbR* disruption, 3’ region |
| SCL286 | CGGCAAGTACTGTGTCCTCA | Nested forward primer for *cnbR* disruption |
| SCL287 | ATGGCAAAGTCGAAGAGGAA | Nested reverse primer for *cnbR* disruption |
| SL391 | TTTGCTCAAGCGTCTCACAT | Primer for *leuA* to confirm *cnbR* disruption |
| SL392 | CATGCAAAAGGCTGACAAGA | Primer for *leuA* to confirm *cnbR* disruption |
| SCL578 | AACCCTTTGGCCTCTCGTAT | RT-PCR primer for *cnbR* |
| SCL579 | AGTCGACATGCTGCGTAATG | RT-PCR primer for *cnbR* |

**References**

1. Nicolas FE, de Haro JP, Torres-Martinez S, Ruiz-Vazquez RM. 2007. Mutants defective in a *Mucor circinelloides* dicer-like gene are not compromised in siRNA silencing but display developmental defects. Fungal Genet Biol 44:504-16.

2. Lee SC, Li A, Calo S, Heitman J. 2013. Calcineurin plays key roles in the dimorphic transition and virulence of the human pathogenic zygomycete *Mucor circinelloides*. PLoS Pathog 9:e1003625.

3. Lee SC, Li A, Calo S, Inoue M, Tonthat NK, Bain JM, Louw J, Shinohara ML, Erwig LP, Schumacher MA, Ko DC, Heitman J. 2015. Calcineurin orchestrates dimorphic transitions, antifungal drug responses and host-pathogen interactions of the pathogenic mucoralean fungus *Mucor circinelloides*. Mol Microbiol 97:844-65.

4. Garcia A, Adedoyin G, Heitman J, Lee SC. 2017. Construction of a recyclable genetic marker and serial gene deletions in the human pathogenic Mucorales *Mucor circinelloides*. G3: Genes, Genomes, Genetics 7:2047-2054.
